# Dexamethasone-Induced Insulin Resistance and Hepatic Metabolic Dysfunction: Modulatory Effects of Montelukast on SGK1-Associated Signaling and NLRP3/Leukotriene-Related Responses

**DOI:** 10.3390/ph19101619

**Published:** 2026-10-12

**Authors:** Ahmad A. Alresheedi, Omnia A. Nour, Dalia H. El-Kashef, Manar A. Nader

**Affiliations:** 1 Department of Pharmacology and Toxicology, Faculty of Pharmacy, Mansoura University, ElGomhoria Street, Mansoura 35516, Egypt; aalresheedi@gmail.com (A.A.A.); omnianour89@mans.edu.eg (O.A.N.); dalia_elkashef@mans.edu.eg (D.H.E.-K.); 2 Faculty of Health Science Technology, Mansoura National University, Gamasa 7731168, Egypt

**Keywords:** montelukast, dexamethasone, insulin resistance, SGK1, AMPK/mTOR signaling, NLRP3 inflammasome, hepatic steatosis, oxidative stress, leukotriene signaling

## Abstract

**Background**: Glucocorticoid-induced metabolic dysfunction and insulin resistance-related abnormalities are important complications associated with dexamethasone (DEX) exposure. This study investigated the protective effects of montelukast (MNT) against DEX-induced metabolic and hepatic disturbances and characterized associated oxidative, inflammatory, leukotriene-related, and insulin-signaling changes in rats. **Methods**: Male Wistar rats were allocated to five groups receiving vehicle, MNT (20 mg/kg), DEX (8 mg/kg), or DEX combined with MNT (10 or 20 mg/kg). Glycemic parameters, oral glucose tolerance, lipid profile, liver function biomarkers, oxidative stress indices, inflammatory mediators, leukotriene-related markers, and hepatic histopathological and ultrastructural changes were assessed. Selected insulin-signaling and metabolic markers were evaluated using immunohistochemistry, ELISA-based assays, and Western blotting. **Results**: DEX administration induced hyperglycemia, hyperinsulinemia, impaired glucose tolerance, increased HOMA2-IR, dyslipidemia, hepatic dysfunction, and histopathological and ultrastructural alterations. These abnormalities were accompanied by oxidative stress and changes in selected molecular markers, including SGK1, AKT, JNK, IRS1, GLUT4, PI3K/FOXO3/PPARγ, GS/PEPCK/GSK, NF-κB/IL-6, NLRP3 abundance, and leukotriene-related markers, including LTD4 and CysLT2, together with alterations in phosphorylation-associated AMPK, PI3K, and ERK1/2 signaling markers. MNT attenuated several DEX-associated metabolic, oxidative, inflammatory, and hepatic abnormalities and modulated these molecular markers. **Conclusions**: MNT attenuated DEX-associated metabolic and hepatic disturbances in rats, accompanied by modulation of selected SGK1-associated insulin-signaling, metabolic, oxidative, inflammatory, and leukotriene-related markers. These findings support further investigation of MNT in experimental glucocorticoid-associated metabolic dysfunction. However, the molecular findings represent associated alterations rather than definitive evidence of direct pathway activation, target engagement, or causal mechanisms.

## 1. Introduction

Insulin resistance (IR) is a state represented by diminished responsiveness to circulating insulin by the peripheral tissues, such as muscles, adipose tissues, and the liver, resulting in impaired glucose uptake and metabolism [1,2]. Although insulin secretion is often enhanced in response to hyperglycemia, insulin-resistant cells continue to respond poorly, resulting in disruption of glucose homeostasis. This impairment disrupts glucose homeostasis and promotes endogenous glucose production in the liver by accelerating glycogenolysis and gluconeogenesis, leading to further aggravated plasma glucose levels and chronic hyperglycemia [3]. Glucocorticoid-induced IR can additionally disrupt hepatic lipid metabolism, promote lipid accumulation and oxidative stress, and ultimately contribute to hepatocellular injury [4,5].

Dexamethasone (DEX), a potent synthetic glucocorticoid, is widely used for its anti-inflammatory and immunosuppressive properties but may produce significant metabolic adverse effects following prolonged exposure [6]. Experimental DEX administration has been used to induce insulin resistance, hyperglycemia, dyslipidemia, and hepatic steatosis and injury [7,8]. DEX-associated hepatic dysfunction may involve disturbances in lipid metabolism, oxidative stress, and cellular homeostasis, including alterations in autophagy and inflammatory signaling [9,10]. Insulin resistance represents an important contributor to these metabolic and hepatic abnormalities, highlighting the potential value of strategies that simultaneously improve insulin sensitivity and attenuate hepatic oxidative and inflammatory injury [11,12].

A comprehensive assessment of chemically induced hepatic injury benefits from integrating biochemical evidence of tissue injury with structural and mechanistic investigations. In this context, Liu et al. applied a toxicological evidence-chain strategy that integrated pathological, metabolic, and mechanistic evidence to characterize the hepatotoxicity of *Dysosma versipellis* [13]. Although the toxicant and experimental context differed from those of the present dexamethasone model, this study highlights the value of integrating complementary levels of evidence to obtain a more comprehensive characterization of chemically induced hepatic injury. Consistent with this approach, the present study combined biochemical, histopathological, ultrastructural, and selected molecular assessments to characterize dexamethasone-associated metabolic and hepatic disturbances.

The relevance of PI3K/AKT/mTOR signaling to hepatic lipid dysregulation is supported by previous experimental evidence. Chu et al. reported that microcystin-LR (MC-LR) aggravated hepatic lipid metabolism disorders in high-fat diet-fed mice and was associated with increased p-PI3K/PI3K, p-AKT/AKT, p-mTOR/mTOR, and SREBP1, implicating the PI3K/AKT/mTOR/SREBP1 axis in hepatic lipid metabolic disturbances [14]. These findings provide a relevant mechanistic context for examining PI3K/AKT-related alterations in glucocorticoid-induced metabolic dysfunction. However, the metabolic insult and experimental setting in the present study differ from those of MC-LR exposure in obese mice. Moreover, the present study assessed selected components of this signaling network rather than the complete phosphorylation/total protein profile or direct SREBP1 regulation. Therefore, these measurements were considered as part of an integrated assessment of insulin- and energy-regulatory signaling rather than as definitive evidence of complete PI3K/AKT/mTOR pathway activation.

Arachidonic acid is metabolized through 5-lipoxygenase (5-LOX)/FLAP to leukotriene (LT)A4. LTA4 can be converted by LTA4 hydrolase to LTB4, which acts principally through BLT1/BLT2 receptors, or is converted through LTC4 synthase to the cysteinyl leukotrienes LTC4, LTD4, and LTE4, which signal mainly through CysLT receptors.

The leukotriene pathway has also emerged as a potential link between inflammation and metabolic dysfunction. Leukotrienes are generated from arachidonic acid through the 5-lipoxygenase (5-LOX) pathway, which produces leukotriene (LT)A4 as a common intermediate. LTA4 is subsequently converted to either LTB4 or the cysteinyl leukotrienes, including LTC4, LTD4, and LTE4. These mediators exert their biological effects through distinct leukotriene receptors, principally the LTB4 receptors BLT1 and BLT2 and the cysteinyl leukotriene receptors CysLT1 and CysLT2 [15]. Activation of these receptor systems has been implicated in leukocyte recruitment, inflammatory signaling, oxidative stress, and tissue injury, processes that may interact with impaired insulin signaling and metabolic dysfunction.

Accordingly, pharmacological targeting of the leukotriene pathway can be approached at different levels, including inhibition of 5-LOX-mediated leukotriene biosynthesis, antagonism of LTB4 receptors, and blockade of cysteinyl leukotriene receptors. These approaches are mechanistically relevant to insulin resistance because leukotriene-mediated inflammatory signaling may influence adipose, hepatic, and systemic metabolic homeostasis. Among these targets, the CysLT1 receptor antagonism is of particular interest because montelukast is a selective CysLT1 receptor antagonist with an established pharmacological profile. Thus, investigation of MNT provides an opportunity to examine whether modulation of cysteinyl leukotriene signaling is associated with attenuation of glucocorticoid-induced metabolic and hepatic abnormalities.

Montelukast (MNT) is a selective antagonist of cysteinyl leukotriene type 1 receptors (CysLT1Rs), which has been reported to effectively inhibit the pro-inflammatory and broncho-constrictive mechanisms of cysteinyl leukotrienes (CysLTs), making it a commonly prescribed agent in the treatment of asthma. It has been suggested that in addition to its receptor-specific actions, MNT also produces secondary antioxidant and anti-inflammatory characteristics that appear to be independent of CysLT1R antagonism [16].

Although several pharmacological approaches have been investigated to counteract glucocorticoid-associated metabolic dysfunction, including agents targeting insulin sensitivity, glucose metabolism, oxidative stress, and inflammatory signaling, the potential of leukotriene-receptor antagonism in DEX-induced insulin resistance and hepatic metabolic injury remains insufficiently characterized. Previous studies investigating MNT have primarily focused on its anti-inflammatory, antioxidant, or hepatoprotective effects in models of LPS-induced, immune-mediated, or chemically induced hepatic injury [17,18,19]. In contrast, the present study addresses DEX-induced insulin resistance as a metabolic–hepatic disorder and examines whether MNT can concurrently modulate glycemic and lipid disturbances, hepatic injury, oxidative stress, inflammation, and interconnected insulin- and energy-regulatory pathways. The specific knowledge gap addressed herein is whether these interconnected molecular domains are concurrently altered during DEX-induced insulin resistance and hepatic dysfunction and whether they are associated with the protective effects of MNT. Accordingly, SGK1 was investigated because of its glucocorticoid responsiveness and potential relevance to insulin resistance, while selected insulin-signaling and energy-regulatory proteins were assessed in relation to hepatic glucose and lipid handling. In parallel, inflammatory, oxidative, and cysteinyl-leukotriene/NLRP3-related markers were examined because of their potential interactions with metabolic and hepatic injury. Importantly, these pathways were investigated as interconnected molecular domains rather than as independent mechanisms, and their concurrent assessment was intended to provide an integrated mechanistic profile rather than establish that any individual pathway is causally required for MNT-mediated protection. Thus, rather than merely mapping downstream biomarkers or reiterating previously reported antioxidant properties, the novelty of this study lies in testing the specific hypothesis that dexamethasone-induced metabolic hepatotoxicity may be closely associated with altered SGK1/NLRP3/mTOR signaling, be hypothesis driven, and montelukast is proposed to offer a novel approach that potentially modulates this pathological cascade. By systematically integrating glucose and lipid homeostasis, hepatic glucose-regulatory enzymes, and energy-regulatory signaling, this work offers a disease-model-specific characterization of the interconnected metabolic alterations associated with dexamethasone exposure and their targeting by montelukast.

## 2. Results

### 2.1. Influence of Montelukast (10 and 20 mg/kg) on Body Weight, Liver Weight, and Liver/Body Weight

**Figure 1:**
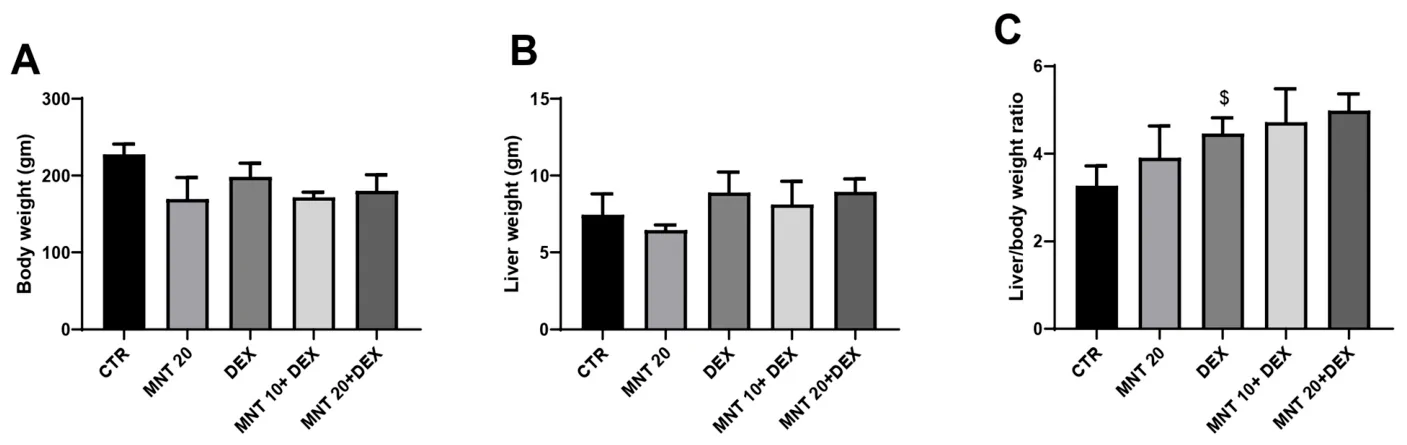
Influence of montelukast (10 and 20 mg/kg) on body weight, liver weight, and liver/body weight. (**A**) Body weight. (**B**) Liver weight. (**C**) Liver/body weight ratio. Data are expressed as mean ± SD (*n* = 4–6 rats/group). Statistical testing was accomplished via one-way ANOVA, then Tukey’s multiple comparisons test for panels (**A**–**C**). ^$^ *p* < 0.05 compared to the CTR group. CTR, control; DEX, dexamethasone; MNT, montelukast.

Regarding body weight and liver weight, there was no significant difference among all groups (Figure 1A,B). DEX administration exhibited a significant (*p* = 0.0254) increase in the liver/body weight ratio compared to the control group. Yet, administration of MNT had no significant effect on the liver/body weight ratio compared to the DEX group (Figure 1C).

### 2.2. Influence of Montelukast (10 and 20 mg/kg) on Glucose Homeostasis

**Figure 2:**
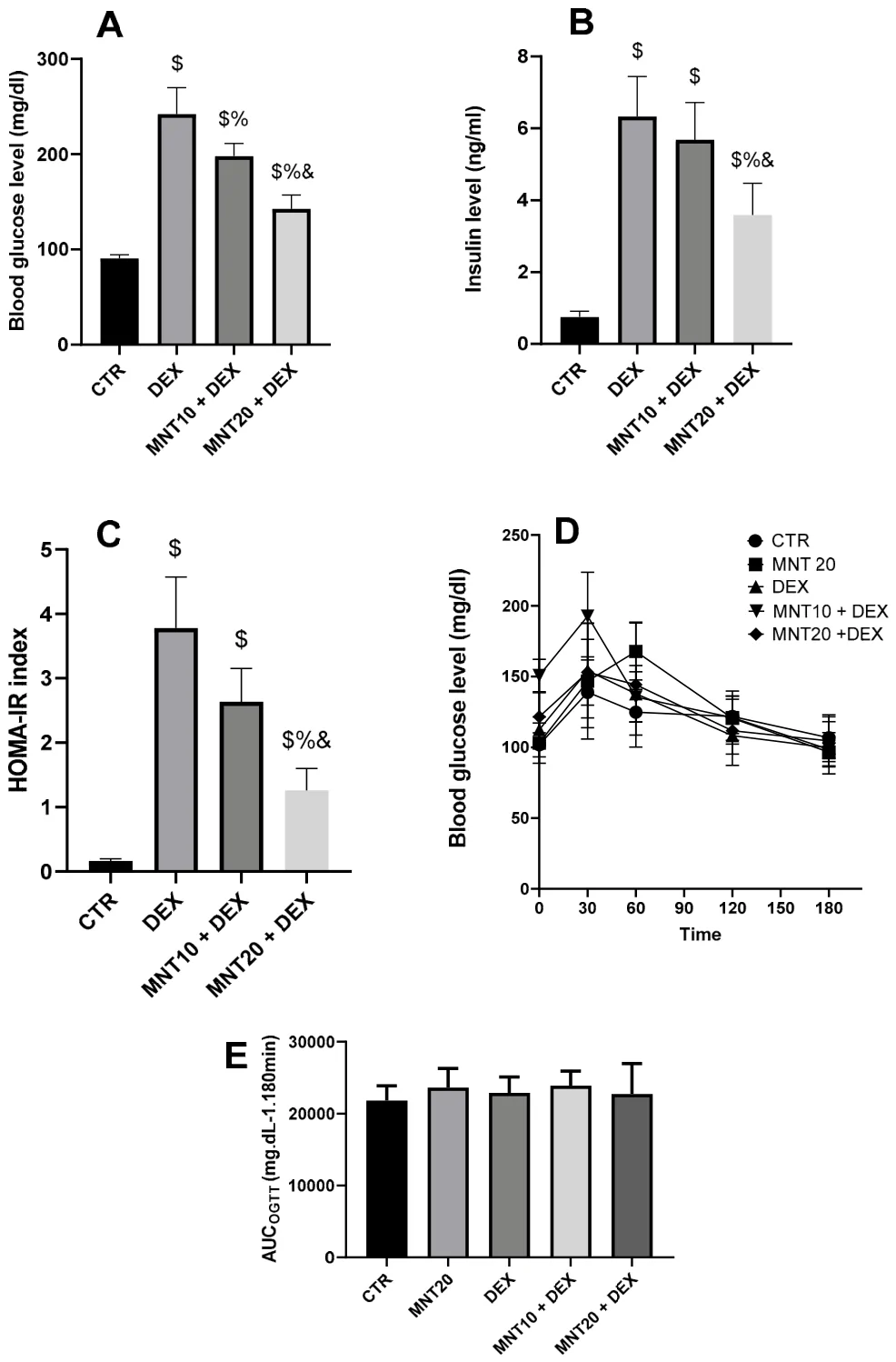
Outcome of MNT on glucose homeostasis in DEX-prompted insulin resistance. (**A**) Fasting blood glucose level. (**B**) Serum insulin level. (**C**) HOMA-IR index. (**D**) OGTT, (**E**) AUC_OGTT._ Data are expressed as mean ± SD (*n* = 4–6 rats/group). Statistical testing was accomplished via one-way ANOVA, then Tukey’s multiple comparisons test for panels (**A**–**C**), and two-way repeated-measures ANOVA, then Tukey’s multiple comparisons test for panel (**D**). $, %, & *p* < 0.05 compared to the CTR, DEX, and MNT 10 + DEX, respectively. CTR, control; DEX, dexamethasone; MNT, montelukast; HOMA-IR, Homeostatic Model Assessment of Insulin Resistance; OGTT, oral glucose tolerance test.

We first evaluated the effects of MNT on DEX-induced disturbances in glycemic homeostasis. Blood glucose levels were significantly higher (*p* < 0.0001) in the DEX group than in the control group (Figure 2A). MNT administration significantly (*p* < 0.0001) reduced serum glucose levels compared with the DEX group (Figure 2A). Consistent with the hyperglycemic state, DEX administration significantly (*p* < 0.0001) increased serum insulin levels (Figure 2B). Co-administration of MNT with DEX reduced the elevated serum insulin levels compared with the DEX group (Figure 2B). The HOMA-IR index was significantly (*p* < 0.0001) increased following DEX administration, indicating increased insulin resistance consistent with changes in β-cell function (Figure 2C). MNT treatment significantly (*p* < 0.0001) attenuated the DEX-induced increase in HOMA-IR in a dose-dependent manner (Figure 2C). Collectively, these findings indicate that MNT improved glycemic homeostasis and it was associated with improvement in surrogate indices of insulin resistance.

Regarding the OGTT, DEX-treated rats exhibited impaired glucose tolerance, as demonstrated by significantly (*p* < 0.05) higher blood glucose levels at the measured time points compared with the control group (Figure 2D). MNT at 10 mg/kg significantly (*p* < 0.05) reduced post-load blood glucose levels compared with the DEX group, indicating improved glucose handling. MNT at 20 mg/kg produced a more pronounced improvement, with glucose levels showing a greater reduction and approaching those of the control group at the later time points of the test (Figure 2D). Regarding AUC_OGTT_, there was no significant difference among all groups (Figure 2E). These findings demonstrate that MNT attenuated the DEX-induced impairment in glucose tolerance. When considered together with fasting glucose, fasting insulin, and HOMA-IR findings, the OGTT results support an overall improvement in glucose homeostasis and surrogate indices of insulin resistance following MNT treatment. However, the OGTT itself does not constitute a direct measure of insulin sensitivity.

### 2.3. Influence of Montelukast (10 and 20 mg/kg) on Liver Function/Structure Alterations

**Figure 3:**
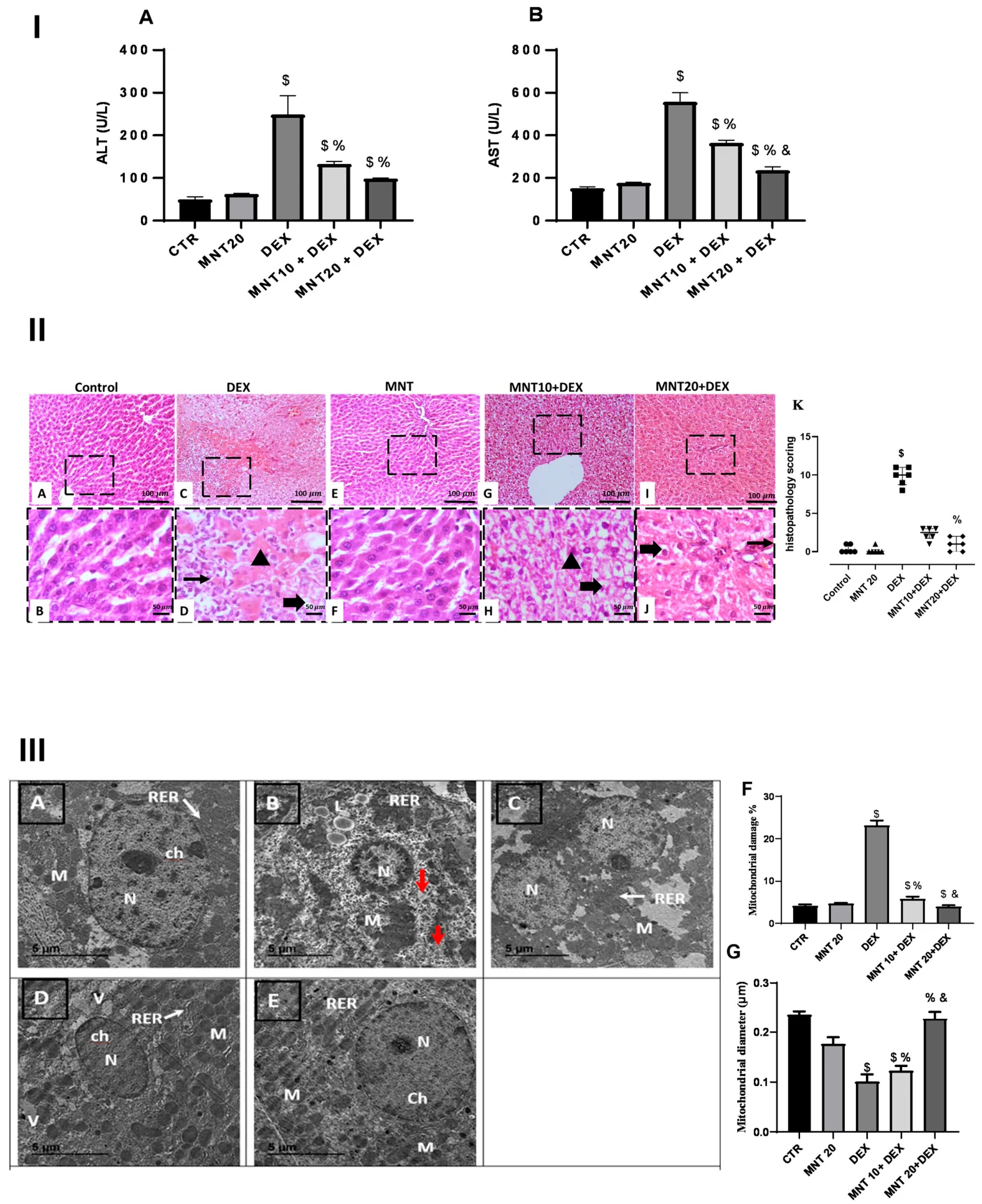
Influence of montelukast (10 and 20 mg/kg) on liver function/structure alterations. (**I**) Impact of montelukast on liver biomarkers in serum: (**A**) ALT and (**B**) AST. Data are articulated as mean ± SD, *n* = 4. $, %, & *p* < 0.05 compared to the CTR, DEX, and MNT 10 + DEX, respectively, via one-way ANOVA. ALT: alanine aminotransferase; AST: aspartate aminotransferase; CTR: control; DEX: dexamethasone; MNT: montelukast. (**II**) Representative photomicrograph of hepatic sections from different treatment groups (low and their corresponding high-power view). (**A**,**B**) Control group. (**C**,**D**) DEX group. (**E**,**F**) MNT20 group. (**G**,**H**) MNT10 + DEX group. (**I**,**J**) MNT20 + DEX. (**K**) The corresponding statistical analysis of the histopathological scoring of hepatic sections of different treatment groups. Thin arrows = inflammation, thick arrows = hepatocellular swelling, arrowheads = hepatocellular necrosis. Image magnification = 100×: Bar 100, 400×: Bar 50. Values are shown as median ± interquartile range; *n* = 6, with Kruskal–Wallis test and Dunn’s adjustment, $, % *p* < 0.05 compared to the CTR and DEX, respectively. (**III**) Transmission electron micrographs of rat liver sections. (**A**) Normal hepatocyte ultrastructure of the control group with a euchromatic nucleus (N), evenly distributed chromatin (ch), mitochondria (M), and well-organized rough endoplasmic reticulum (RER). (**B**) Hepatocytes have shown drastic ultrastructural disturbances in DEX-treated group. Loss of normal cell architecture was noted, with irregularity observed in the nuclei (N), and disordered cytoplasmic components. They showed many lipid droplets (L), mitochondria (M) were degenerative and electrondense, nuclei frequently pyknotic and showed vacuolization of the RER was significantly dilated. Vesicular cytoplasm was also demonstrated (red arrow). (**C**) In the MNT20 group, hepatocytes contained large, spherical nuclei (N) and had normal morphology. The cytoplasm showed well-preserved mitochondria and regularly arranged RER similar to the control group. (**D**) In the MNT10 + DEX group, N of hepatocytes displayed mildly increased ch at the peripheral. Most mitochondria appeared relatively normal except for some variable size (white arrows), and focal dilatation of RER. Cytoplasmic vacuolation (V) persisted. (**E**) Near-complete restoration of cellular ultrastructure in MNT20 + DEX-treated hepatocytes. Nuclei (N) were almost normal and many mitochondria (M) with preserved morphology in cytoplasm, being similar to the group of control. (**F**) Quantitative scoring of mitochondrial damage percentage. (**G**) Quantitative scoring mitochondrial diameter (µm). Data are articulated as mean ± SD, *n* = 6. $, %, & *p* < 0.05 compared to the CTR, DEX, and MNT 10 + DEX, respectively, via one-way ANOVA.

The effect of MNT was evaluated against DEX-induced liver function markers. It was found that rats treated with DEX demonstrated considerable (*p* < 0.0001) elevation in levels of both ALT and AST compared to the normal control group (Figure 3IA,IB). However, administration of MNT at 10 and 20 mg/kg significantly (*p* < 0.0001) reduced the ALT and AST levels matched to the DEX set. Interestingly, there was more reduction in ALT and AST at the higher dose than at the lower dose of MNT that was statistically significant by only AST. Importantly, MNT alone did not raise ALT and AST levels to any significant level (Figure 3IA,IB).

These biological outcomes were further supported by histopathological results, which presented the following: The control group displayed normal histology of hepatic parenchyma. DEX rats expressed severe hepatocellular swelling with numerous vacuoles and multifocal to coalescing extensive hepatocellular necrosis illustrated by hyper-eosinophilic cytoplasm with pyknotic or karyolitic nuclei surrounded and infiltrated with dense neutrophilic, lymphoplasmacytic, and phagocytic cells. MNT showed apparently normal hepatic parenchyma. MNT10 + DEX showed moderate hepatocellular swelling with scattered necrotic hepatocytes. MNT20 + DEX showed mild hepatocellular swelling and vacuolation with little periportal inflammation (Figure 3II). Scoring of liver macro-vesicular steatosis and inflammation displayed substantially greater scores of liver inflammation and steatosis in the DEX group compared to controls but significantly less in the entire management groups, with little alterations between them (Figure 3II).

Regarding TEM outcomes, control group liver micrographs displayed normal euchromatic nucleus hepatocytes, evenly distributed chromatin, and well-conserved mitochondria in the cytoplasm along with a typically arranged rough endoplasmic reticulum. The DEX group demonstrated marked ultrastructural alterations; hepatocytes exhibited abnormal cellular architecture, irregular pyknotic nuclei, and disorganized cellular constituents; various lipid droplets, degenerated and electron-dense mitochondria, prominently dilated rough endoplasmic reticulum, and noticeably vacuolated cytoplasm were also observed. The MNT20 group displayed large, normal, spherical nuclei in hepatocytes with the cytoplasm-enclosed well-conserved mitochondria and typically arranged rough endoplasmic reticulum. The MNT10 + DEX group showed hepatocytes with nuclei exhibiting a slight increase in peripheral chromatin; numerous normally shaped mitochondria of variable sizes were observed, along with focal dilation of the rough endoplasmic reticulum and still vacuolated cytoplasm. The MNT20 + DEX group showed near-complete restoration of hepatic cellular ultrastructure comparable to the control group, with a nearly regular nucleus and cytoplasm containing well-preserved mitochondria (Figure 3III).

The concordance between the biochemical and morphological findings provides a more comprehensive assessment of the hepatic effects of MNT.

### 2.4. Effect of Montelukast (10 and 20 mg/kg) on Lipid Profile

**Figure 4:**
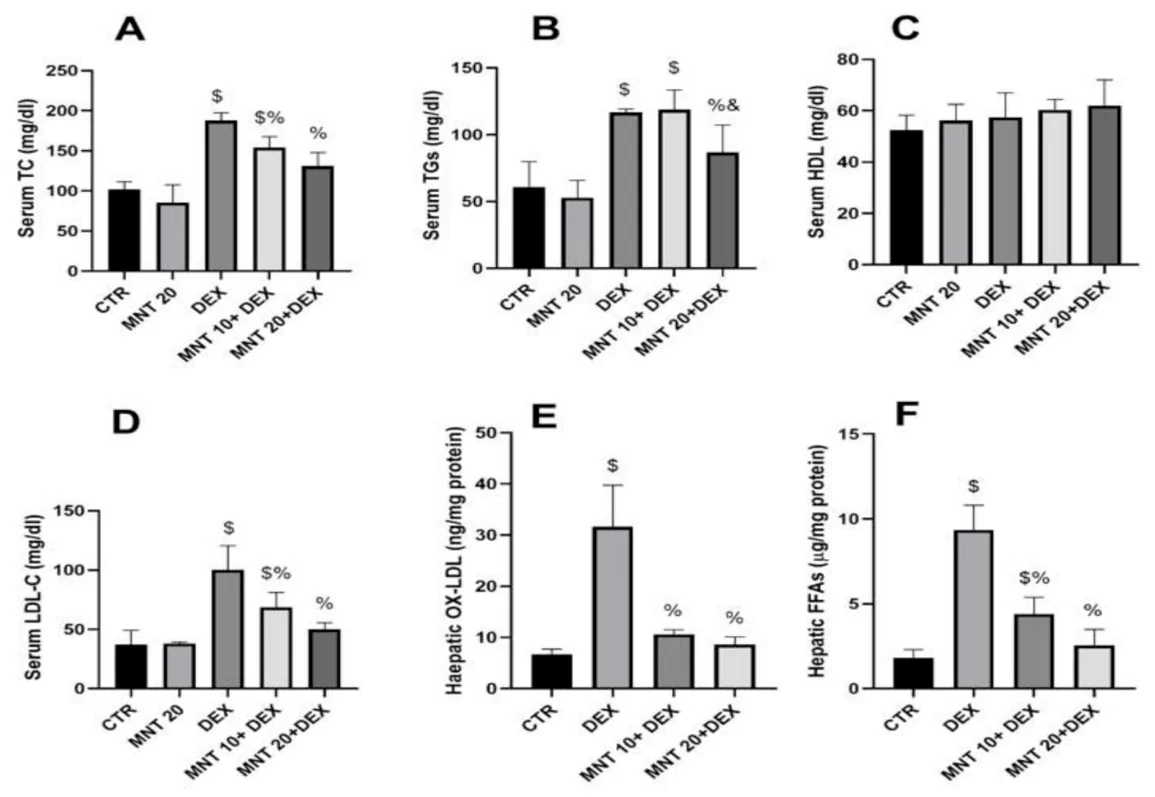
Effect of MNT on DEX-induced dyslipidemia. (**A**) Serum TC. (**B**) Serum TGs. (**C**) Serum HDL-C. (**D**) Serum LDL-C. (**E**) Hepatic OX-LDL. (**F**) FFAs. results are conveyed as mean ± SD (*n* = 4–6 rats/group). Statistical examinations were carried out via one-way ANOVA. $, %, & *p* < 0.05 vs. CTR, DEX, and MNT 10 + DEX groups, respectively. DEX, dexamethasone; MNT, montelukast; TC, total cholesterol; TGs, triglycerides; HDL, high-density lipoprotein cholesterol; LDL-C, low-density lipoprotein cholesterol; OX-LDL, oxidized low-density lipoprotein; FFAs, free fatty acids.

Dyslipidemia is one of the major consequences of DEX use. Hence, we examined the status of lipid contents, including TC, HDL-C, LDL-C, OX-LDL, TG, and FFA following DEX monotherapy and combination of DEX with MNT in two doses. It was found that DEX markedly (*p* < 0.05) elevated lipid contents except HDL-C, which showed no significant change (Figure 4A–F). Conversely, combinatory administration of MNT and DEX reverted the hyperlipidemic effects caused by DEX single therapy (Figure 4A–F). It was noted that co-administration of MNT and DEX led to a significant (*p* < 0.0001) reduction of TC, LDL-C, OX-LDL, TG, and FFA. It was also observed that a higher dose of MNT substantially (*p* < 0.0001) lowered the effects compared to the lower dose. Although, co-administration of MNT at 20 mg/kg significantly (*p* = 0.0194) reduced TG levels, whereas MNT at 10 mg/kg did not show a statistically significant reduction compared to the DEX group (Figure 4B). Serum HDL-C levels showed a slight, non-significant elevation in MNT-treated groups compared with the DEX group (Figure 4C). These results highlight the ameliorating effects of MNT on the dyslipidemic actions of DEX.

### 2.5. Influence of MNT (10 and 20 mg/kg) on SGK1

**Figure 5:**
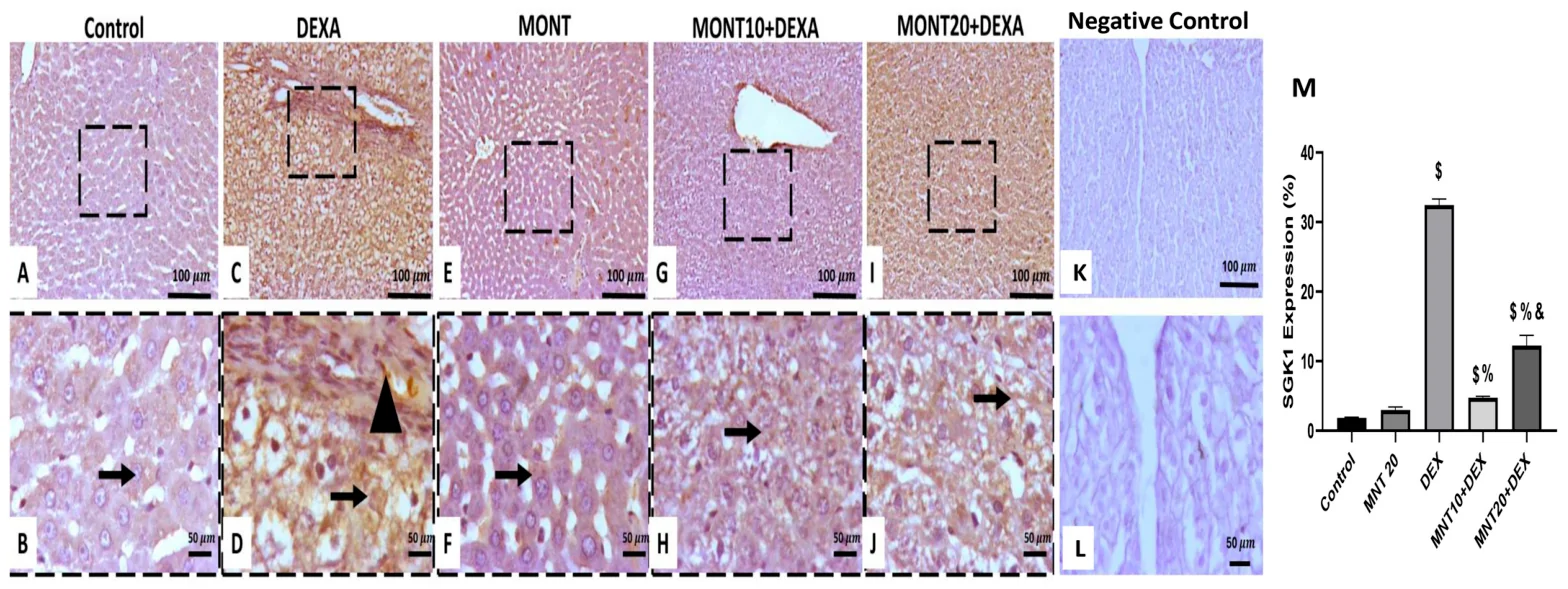
Representative IHC expression and localization of SGK1 in hepatic sections of different treatment groups (low and their corresponding high-power view). (**A**,**B**) Control group. (**C**,**D**) DEX group. (**E**,**F**) MNT20. (**G**,**H**) MNT10 + DEX. (**I**,**J**) MNT20 + DEX showing moderate faint cytoplasmic expression in ballooned hepatocytes. (**K**,**L**) Negative control. (**M**) SGK1% expression. Panels (**A**,**C**,**E**,**G**,**I**,**K**) were captured at ×100 magnification (scale bar = 100 μm), while panels (**B**,**D**,**F**,**H**,**J**,**L**) represent higher magnification (×400; scale bar = 50 μm) of the boxed areas. Thin arrows = positive hepatocytes, arrowhead = positive fibrotic tissue with inflammatory cells. Image magnification = 100×: Bar 100, 400×: Bar 50. The corresponding statistical analysis of SGK1 expression in hepatic sections of different treatment groups. Values are shown as mean ± SD, with one-way ANOVA, $, %, & *p* < 0.05 vs. CTR, DEX, and MNT 10 + DEX groups, respectively.

IHC expression and localization of SGK1 in hepatic slices from diverse treatment groups disclosed the following: The control group showed weak cytoplasmic expression in a few hepatocytes. DEX showed diffuse intense membranous and cytoplasmic expression with variable nuclear expression in ballooned hepatocytes, together with positively stained fibroblasts and inflammatory cells. The MNT20 group showed scattered weak cytoplasmic immunostained hepatocytes. MNT10 + DEX showed mild faint cytoplasmic expression in mildly swollen hepatocytes. MNT20 + DEX showed moderate faint cytoplasmic expression in ballooned hepatocytes (Figure 5A–J). A statistical calculation of % of positively stained areas disclosed immune expression of SGK1 that was meaningfully (*p* < 0.0001) enhanced in the DEX group when compared to the control group. MNT (10 and 20 mg/kg) + DEX dose-dependently decreased hepatic percent immune expression compared to the DEX group (Figure 5K).

### 2.6. Influences of MNT on p-AMPK, p-PI3K and p-ERK1/ERK2 Protein Expressions

**Figure 6:**
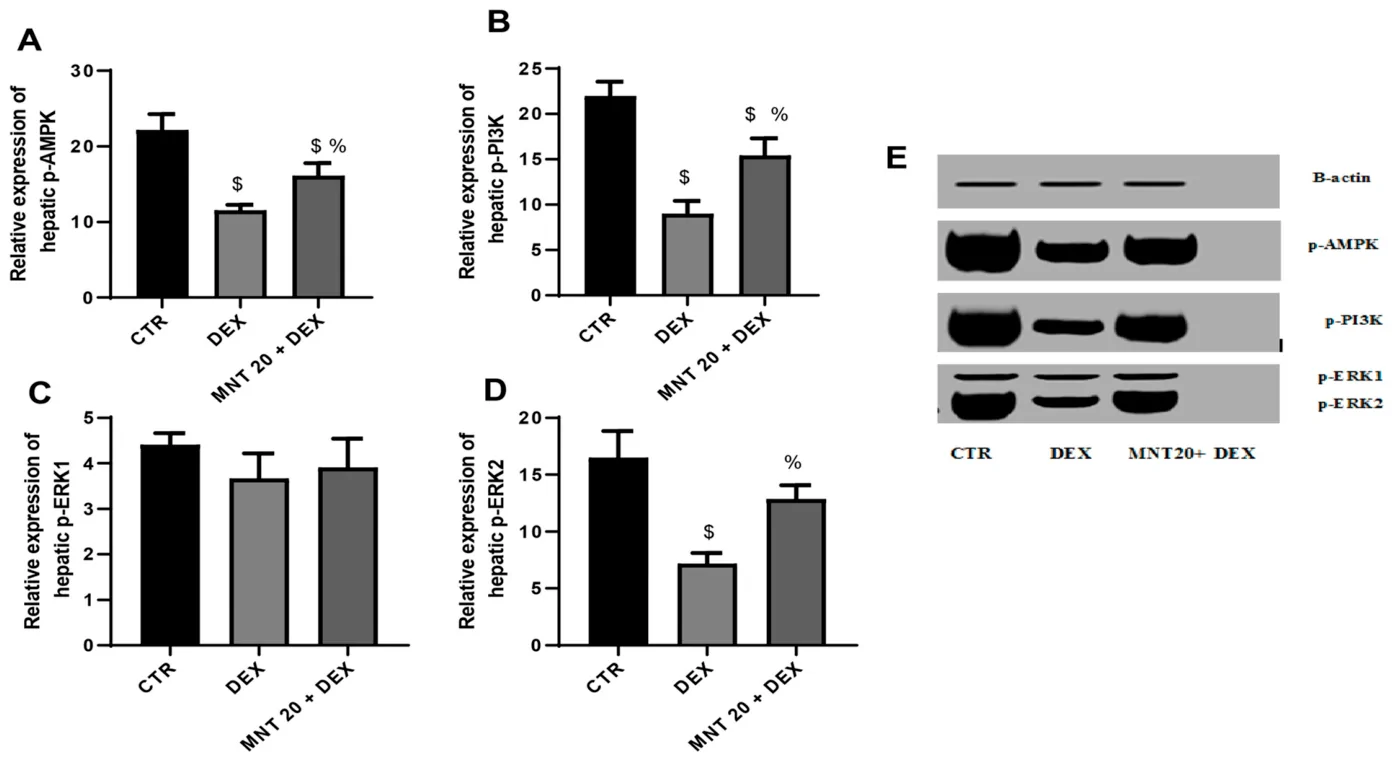
Influence of MNT on (**A**) relative expression of p-AMPK; (**B**) relative expression of p-PI3K; (**C**) relative expression of p-ERK1, (**D**) relative expression of ERK2, and (**E**) representative Western blot bands. Data are conveyed as mean ± SD, *n* = 3. $, % *p* < 0.05 vs. CTR and DEX, correspondingly, via one-way ANOVA. CTR: control; DEX: dexamethasone; p-AMPK: phosphorylated AMP-activated protein kinase, p-PI3K: phosphorylated Phosphoinositide 3-Kinase, p-ERK: phosphorylated extracellular signal-regulated kinase.

To further characterize metabolic signaling alterations, hepatic phosphorylation-associated signaling components were also assessed. Compared with the control group, DEX administration significantly (*p* < 0.0001) reduced hepatic p-AMPK, p-PI3K, and p-ERK2 expression. In contrast, MNT treatment significantly (*p* < 0.0001) increased these phosphorylation-associated markers compared with the DEX group (Figure 6A,B,D). Collectively, these findings indicate that MNT modulated selected insulin- and energy-regulatory signaling components in association with the improvement in metabolic abnormalities. Also, Western blot analysis indicated significant restoration of p-ERK2 expressions following MNT treatment, whereas p-ERK1 showed no significant alterations across the experimental groups (Figure 6C). Therefore, the observed changes in ERK phosphorylation are primarily attributable to the p-ERK2 isoform. Nevertheless, these changes should not be interpreted as definitive evidence of restoration of the complete insulin signaling pathway, as isoform-specific phospho-AKT, IRS-1 phosphorylation, JNK activity, GLUT4 membrane translocation, and functional glucose uptake were not directly assessed.

### 2.7. Effect of MNT (10 and 20 mg/kg) on Insulin-Related Signaling Protein

**Figure 7:**
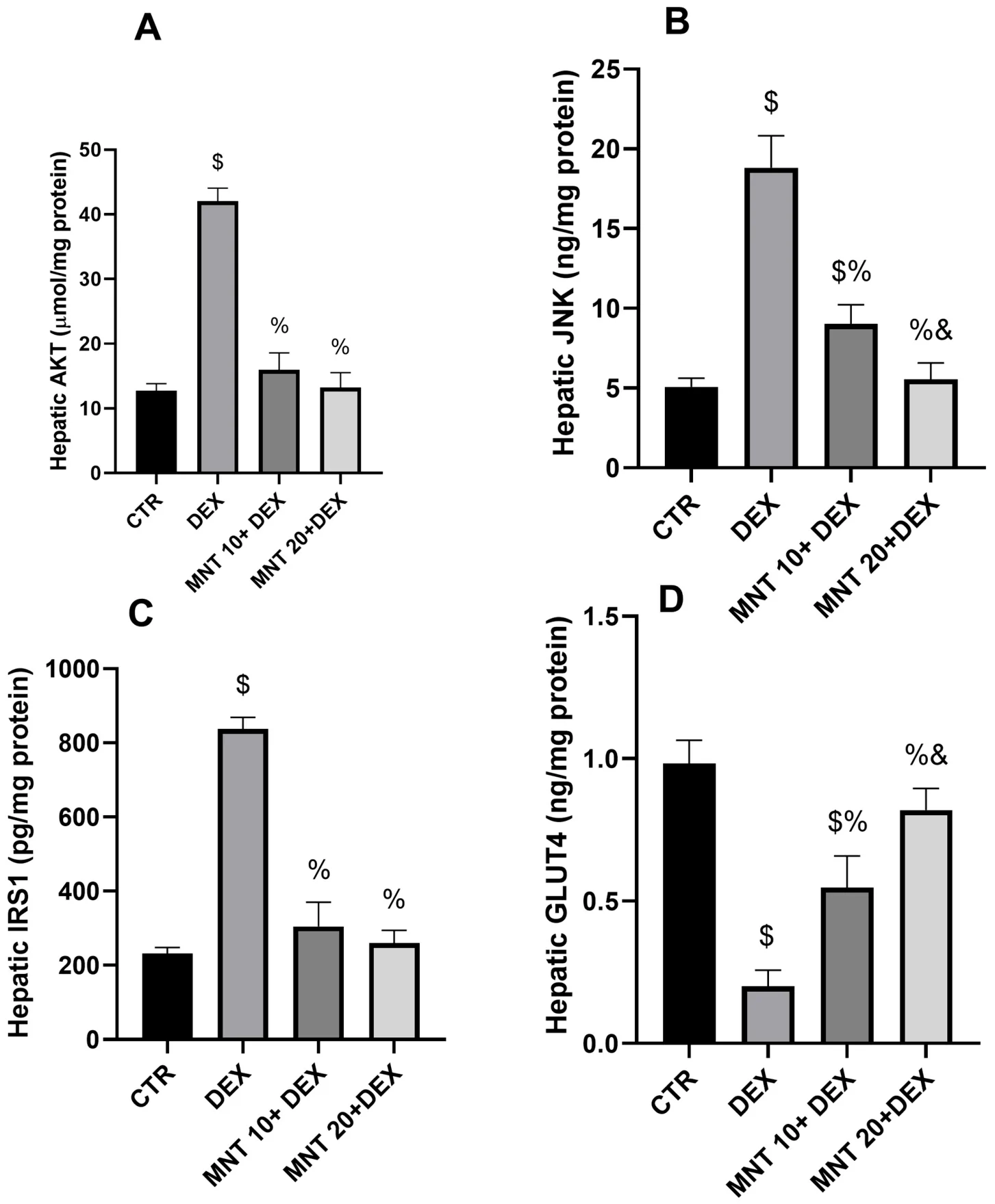
Consequence of MNT on hepatic insulin signaling biomarkers in DEX-provoked IR. (**A**) AKT level. (**B**) JNK level. (**C**) IRS-1 level. (**D**) GLUT4 level. Results are presented as mean ± SD (*n* = 6 rats/group). Statistics were performed via one-way ANOVA followed by Tukey’s multiple comparisons test. $ *p* < 0.05 versus the control (CTR) group; $, %, & *p* < 0.05 compared to the CTR, DEX, and MNT 10 + DEX, respectively. DEX, dexamethasone; MNT, montelukast; AKT, protein kinase B; JNK, c-Jun N-terminal kinase; IRS-1, insulin receptor substrate-1; IR, insulin resistance; GLUT4, glucose transporter type 4.

We explored selected insulin-signaling-related proteins to characterize molecular alterations associated with DEX-induced metabolic dysfunction and the response to MNT treatment. DEX significantly increased hepatic total AKT protein abundance (*p* < 0.0001), whereas both MNT doses attenuated this DEX-associated increase toward control levels (Figure 7A). Similarly, hepatic IRS-1 protein abundance was significantly (*p* < 0.0001), increased in the DEX group and was attenuated following MNT administration (Figure 7C). Hepatic total JNK protein abundance was also significantly (*p* < 0.0001) increased following DEX administration and was reduced following MNT treatment in a dose-related manner (Figure 7B). Hepatic total GLUT4 protein abundance was significantly (*p* < 0.0001) reduced following DEX treatment and was increased following MNT co-administration (Figure 7D). The higher MNT dose produced a greater attenuation of the DEX-associated reduction in GLUT4 abundance. Collectively, these findings indicate that MNT modulated the abundance of several proteins related to insulin signaling and glucose handling. However, because these measurements represent total protein abundance, they do not directly establish changes in protein activation or functional insulin signaling. In particular, the GLUT4 findings reflect total hepatic protein abundance and should not be interpreted as evidence of increased GLUT4 translocation to the plasma membrane.

### 2.8. Influence of MNT (10 and 20 mg/kg) on AMPK Protein Levels and mTOR Protein Levels Protein Expressions

**Figure 8:**
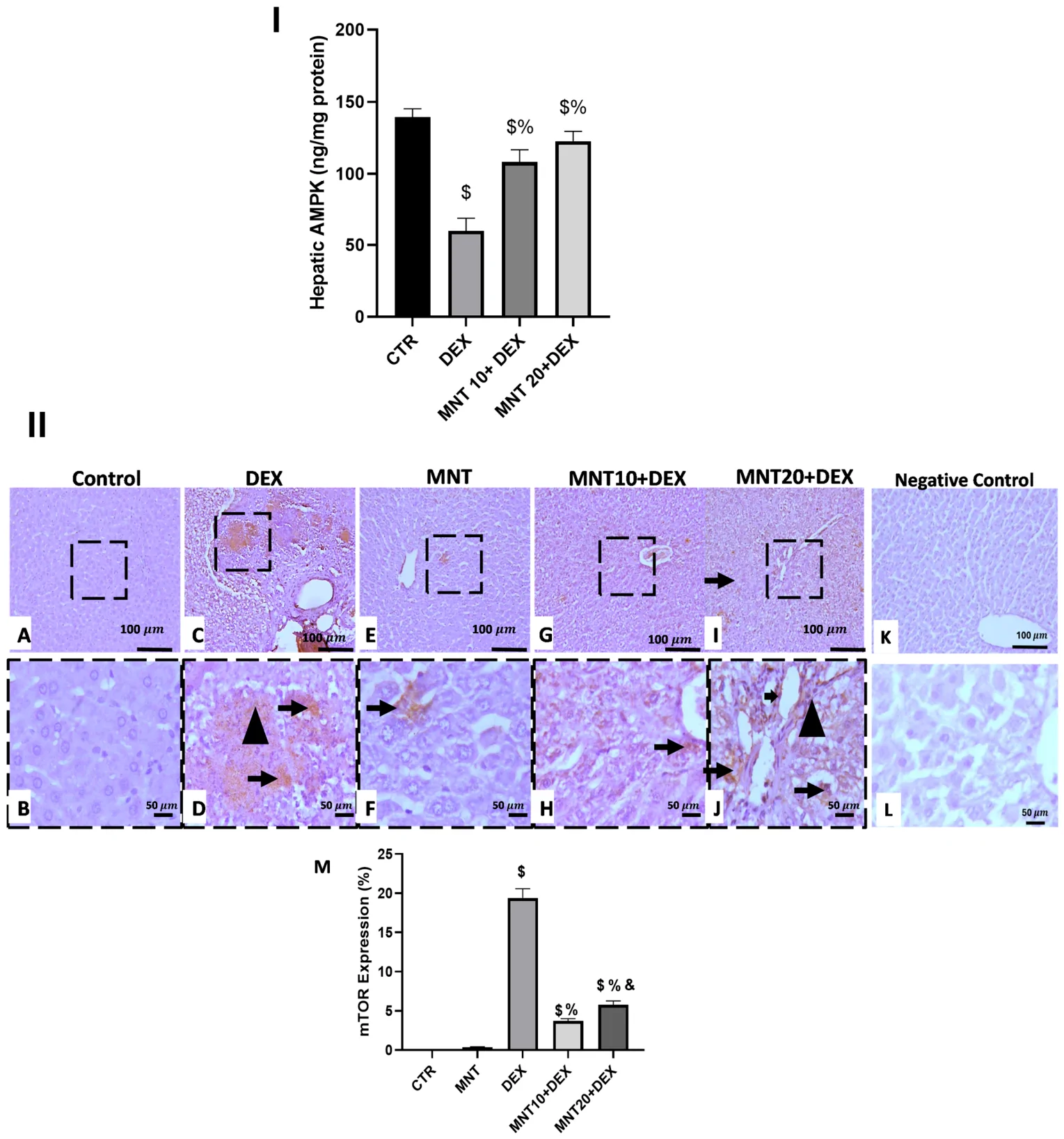
Effect of MNT on hepatic AMPK and mTOR levels in DEX-stimulated IR. (**I**) AMPK level. Data are expressed as mean ± SD (*n* = 4 rats/group). Statistical examination was performed via one-way ANOVA. $, % *p* < 0.05 compared to the CTR and DEX, respectively. (**II**) Representative IHC photomicrographs of hepatic slices of mTOR expression. (**A**,**B**) The control group showed minimal mTOR immunoreactivity. (**C**,**D**) The DEX-treated group exhibited noticeable positive mTOR immunostaining (brown color; arrows) in hepatocytes, with obvious expression (arrowhead). (**E**,**F**) The MNT-treated group showed immunostaining compared to the control group. (**G**,**H**) The MNT 10 + DEX group showed reduced mTOR immunoreactivity compared to the DEX group. (**I**,**J**) MNT 20 + DEX showed a further decrease in mTOR expression to near the control pattern. Panels (**A**,**C**,**E**,**G**,**I**) were captured at ×100 magnification (scale bar = 100 μm), while panels (**B**,**D**,**F**,**H**,**J**) represent higher magnification (×400; scale bar = 50 μm) of the boxed areas. (**K**,**L**) Hepatic mTOR-positive area (%). Data are expressed as mean ± SEM (*n* = 4 rats/group). Statistical testing was performed via one-way ANOVA. $, %, & *p* < 0.05 compared to the CTR, DEX, and MNT 10 + DEX, respectively. (**M**) mTOR expression. DEX, dexamethasone; IR, insulin resistance; MNT, montelukast; mTOR, mammalian target of rapamycin; AMPK, AMP-activated protein kinase.

Following the study of AKT and related factors, we were impelled to ascertain the influence on AMPK. The results exhibited that AMPK was markedly (*p* < 0.0001) dampened by injection of DEX in rats (Figure 8I). On the other hand, MNT raised the AMPK expression near the control group of rats.

Expression of mTOR in hepatic slices of various groups demonstrated the following: The control group showed faint cytoplasmic expression in hepatocytes. The DEX group showed multifocal moderate to high granular expression in ballooned hepatocytes. MNT20 showed few individual cytoplasmic immune-stained hepatocytes. MNT10 + DEX showed few cytoplasmic expressions in vacuolated hepatocytes and surrounding invading inflammatory cells. MNT20 + DEX showed mild scattered cytoplasmic expression in vacuolated hepatocytes with biliary epithelia cytoplasmic staining and few positively stained periportal inflammatory cells (Figure 8II). The statistical estimation of % positive stained areas disclosed that hepatic percent immune expression of mTOR was significantly (*p* < 0.0001) enhanced in the DEX group when compared to the control group. Administration of MNT in both doses with DEX dose-dependently decreased hepatic percent immune expression compared to the DEX group (Figure 8II).

These findings indicate that MNT modulated selected components of the hepatic AMPK/mTOR signaling pathway. The concurrent normalization of AMPK, p-AMPK, and mTOR expression with the improvement in glucose and lipid abnormalities is consistent with involvement of AMPK/mTOR-associated energy-regulatory signaling in the metabolic effects of MNT. However, because pathway-specific inhibition or activation was not performed, these findings should be interpreted as an association rather than evidence that AMPK/mTOR signaling is directly responsible for the observed metabolic improvements.

### 2.9. Effect of MNT (10 and 20 mg/kg) on Metabolic Alteration Concerning ERK and Its Related Molecules

**Figure 9:**
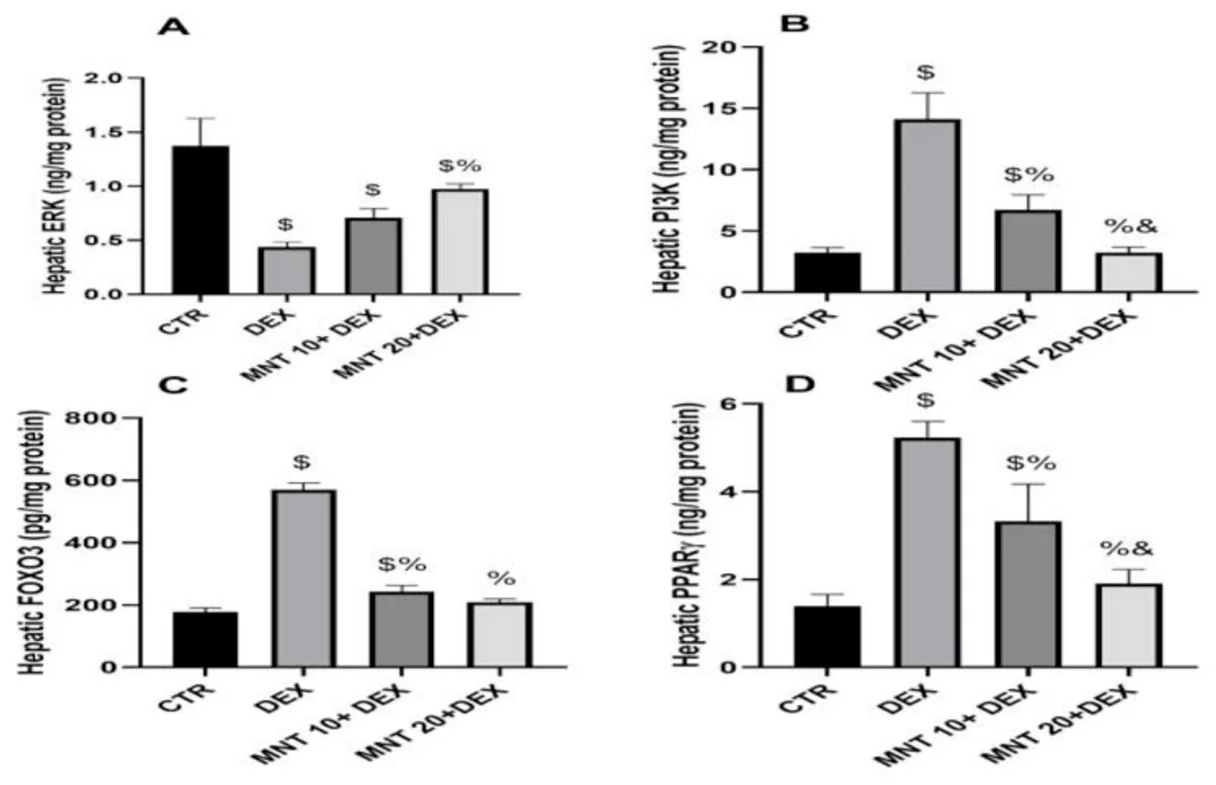
Influences of MNT treatment on hepatic ERK and related protein alterations. (**A**) ERK, (**B**) PI3K, (**C**) FOXO3, and (**D**) PPARγ. Data are extracted as mean ± SD, *n* = 4, and analyzed via one-way ANOVA. $, %, & *p* < 0.05 compared to the CTR, DEX, and MNT 10 + DEX, respectively. CTR: control; DEX, dexamethasone; ERK, extracellular signal-regulated kinase; FOXO3, Forkhead Box O3; PI3K—Phosphoinositide 3-Kinase and PPARγ, peroxisome proliferator-activated receptor gamma.

To explore the expanded role of DEX-provoked metabolic alterations and the efficient MNT actions, we investigated ERK and its related molecules. The hepatic ERK level (measured by ELISA) was markedly (*p* < 0.0001) diminished in DEX-injected rats, in contrast to the control group (Figure 9A). Conversely, MNT at 10 and 20 mg/kg showed a significant (*p* < 0.0001) rise in the levels of ERK matched to the DEX-treated group. The total PI3K protein abundance (measured by ELISA) was markedly (*p* < 0.0001) augmented in the DEX group of rats compared to the normal group. Meanwhile, MNT treatment resulted in a marked (*p* < 0.0001) reduction in total PI3K levels (Figure 9B). Additionally, matched to MNT10 + DEX, MNT20 + DEX showed a significantly (*p* < 0.05) greater reduction.

Moreover, hepatic FOXO3/PPARγ levels were evaluated in the current research, and it was found that DEX considerably elevated FOXO3/PPARγ as compared to the control rat group (Figure 9C,D). However, levels of FOXO3/PPARγ were lowered to significant levels (*p* < 0.0001) upon MNT treatment in both incremental doses. Additionally, matched to MNT10 + DEX, MNT20 + DEX showed a significantly (*p* = 0.0087) greater reduction in hepatic FOXO3/PPARγ levels.

Collectively, these findings indicate that MNT modulated selected components of the ERK/PI3K/FOXO3/PPARγ signaling network in association with the improvement in DEX-induced metabolic abnormalities.

### 2.10. Effect of MNT (10 and 20 mg/kg) on GS, PEPCK, SREBP1, and GSK

**Figure 10:**
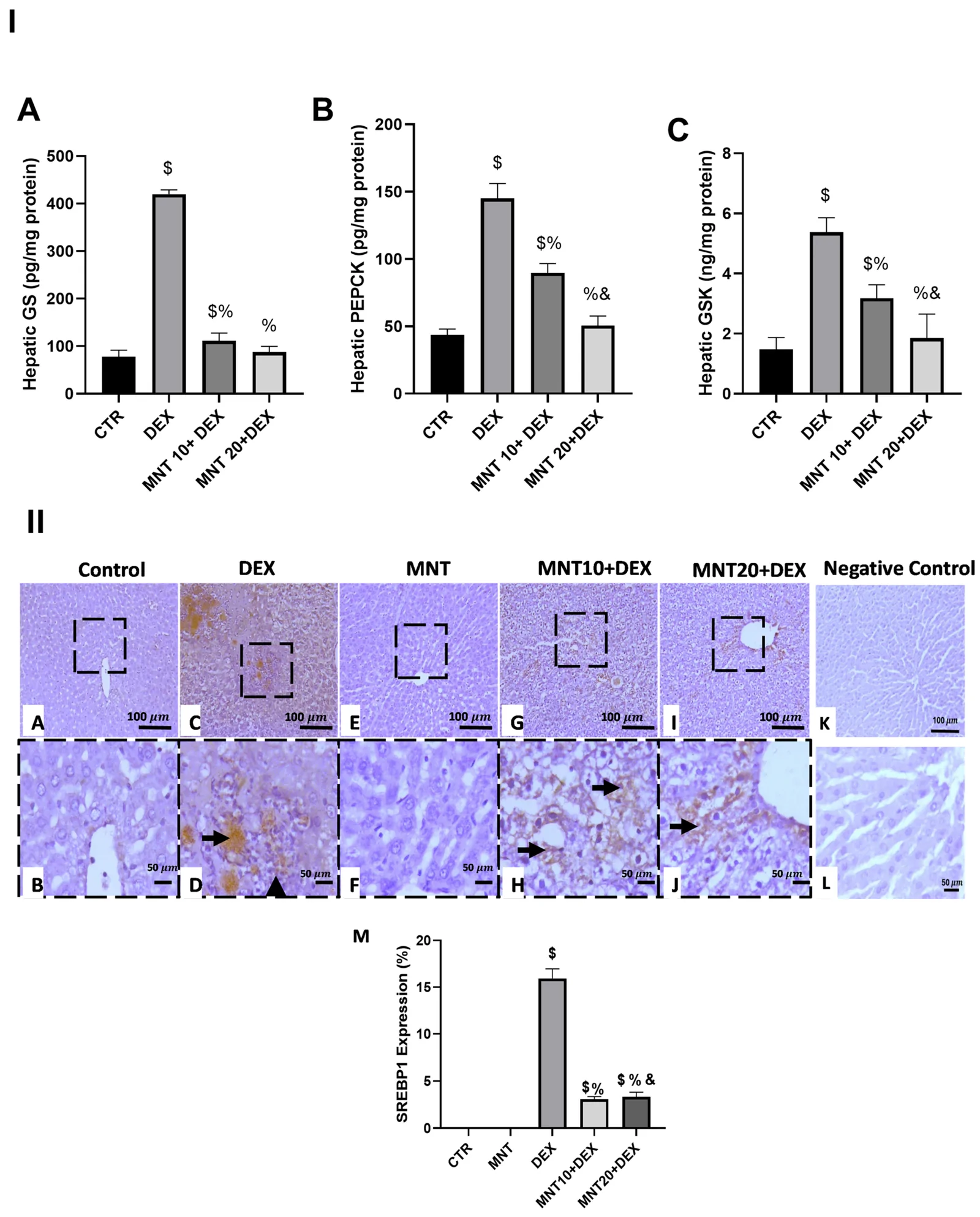
Effect of MNT on hepatic biomarkers of glycogen synthesis/gluconeogenesis in DEX-provoked metabolic alterations. (**I**): (**A**) GS, (**B**) PEPCK, (**C**) GSK. Data are expressed as mean ± SD. $ *p* < 0.05 vs. CTR group, $, %, & *p* < 0.05 compared to the CTR, DEX, and MNT 10 + DEX, respectively using one-way ANOVA. *n* = 4. (**II**) IHC and quantitative analysis of hepatic SREBP1 expression among experimental groups. (**A**–**J**) Representative immunohistochemical microphotographs of liver sections: (**A**,**B**) CTR group presenting baseline/minimal SREBP1 immunostaining. (**C**,**D**) DEX group displaying a significant upregulation and intense positive immunoreactivity for SREBP1 (arrows/arrowheads). (**E**,**F**) MNT group alone displaying baseline expression near control. (**G**,**H**) MNT10 + DEX group revealing an obvious lessening in SREBP1 expression compared to DEX group. (**I**,**J**) MNT20 + DEX group representing significant diminution of DEX-stimulated SREBP1 expression. (**K**,**L**) Negative control. (**M**) Quantitative evaluation of SREBP1 expression area percentage (%). Scale bars represent 100 (low power: (**A**,**C**,**E**,**G**,**I**,**K**) and 50 (high power/insets: (**B**,**D**,**F**,**H**,**J**,**L**). Data is presented as mean ± SD (*n* = 4) using one-way ANOVA. $, %, & *p* < 0.05 compared to the CTR, DEX, and MNT 10 + DEX, respectively. CTR, control; DEX, dexamethasone; GS, glycogen synthase; GSK, glycogen synthase kinase; PEPCK; phosphoenolpyruvate carboxykinase; SREBP1, sterol regulatory element-binding protein 1.

The hepatic levels of GS, PEPCK, and GSK were markedly (*p* < 0.0001) augmented in the DEX treatment group compared to the control group (Figure 10I). In contrast, treatment with MNT led to an effective reduction in levels of GS, PEPCK, and GSK in a dose-incremental manner (*p* < 0.0001). Regarding PEPCK and GSK, the higher doses of MNT showed more prominent (*p* < 0.05) reduction than the lower doses (Figure 10I).

Concurrently, the hepatic IHC expression of SREBP1 disclosed the following: The control group showed negative expression of SREBP1 in hepatocytes. The DEX group showed multifocal patches of strong expression in degenerated and necrotic hepatocytes with little positivity in the admixed inflammatory cells. MNT20 showed negative immune-stained hepatocytes. MNT10 + DEX showed scattered mild weak cytoplasmic expression in vacuolated hepatocytes. MNT20 + DEX showed weak focal cytoplasmic expression in the perivascular vacuolated hepatocytes (Figure 10II). A statistical analysis of % positive stained areas disclosed that in the DEX group, the hepatic % positive SREBP1 stained area was significantly enhanced when compared to the control group. MNT (10 and 20 mg/kg) + DEX administration decreased hepatic percent immune expression compared with the DEX group (Figure 10II).

### 2.11. Effect of Montelukast (10 and 20 mg/kg) on NF-κB/IL-6

**Figure 11:**
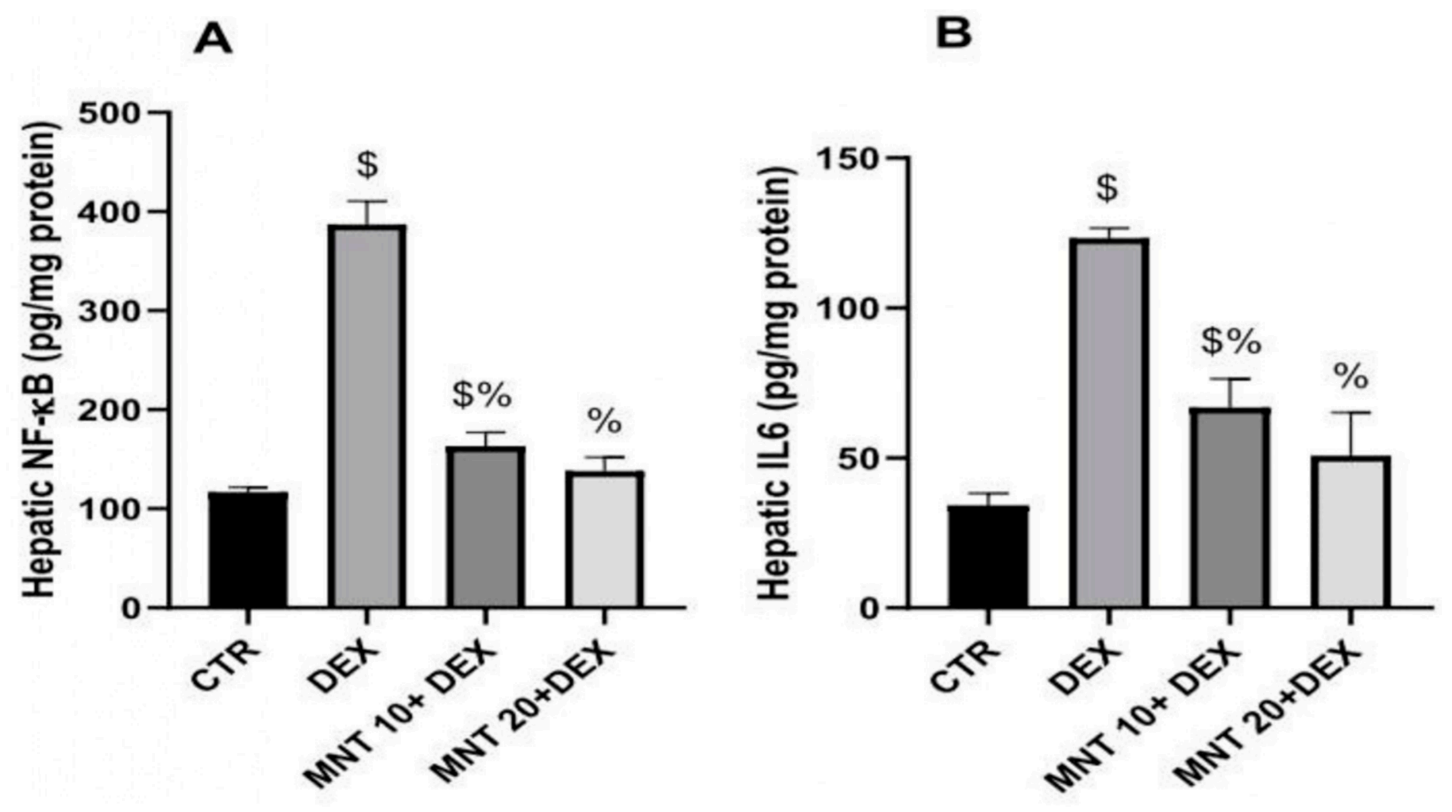
Effect of MNT on DEX-stimulated hepatic inflammatory markers. (**A**) NF-κB and (**B**) IL6. Data are expressed as mean ± SD using one-way ANOVA, *n* = 4. $, % *p* < 0.05 compared to the CTR and DEX, respectively. CTR, control; DEX, dexamethasone; IL-6, interleukin 6; MNT, montelukast; NF-κB, nuclear factor Kappa B.

We further examined the expressions of inflammatory markers, including NF-κB and IL-6 to confirm the inflammatory alterations linked to DEX and MNT treatments. It was noted that DEX considerably (*p* < 0.0001) boosted the expression of both NF-κB and IL-6, highlighting the inflammation induced by this steroidal agent (Figure 11A,B). However, upon application of MNT 10 and 20 with DEX, the abundance of both NF-κB and IL-6 was evidently (*p* < 0.0001) downregulated in a dose-dependent pattern (Figure 11A,B). Therefore, these outcomes suggest that DEX induces inflammatory changes in rats via recruitment of NF-κB and IL-6, while MNT exerts anti-inflammatory effects by diminishing the levels of both inflammatory mediators. The statistical analysis also showed that the higher dose produced more anti-inflammatory actions compared to the lower dose of MNT.

### 2.12. Influences of Montelukast (10 and 20 mg/kg) on Oxidative Status

**Figure 12:**
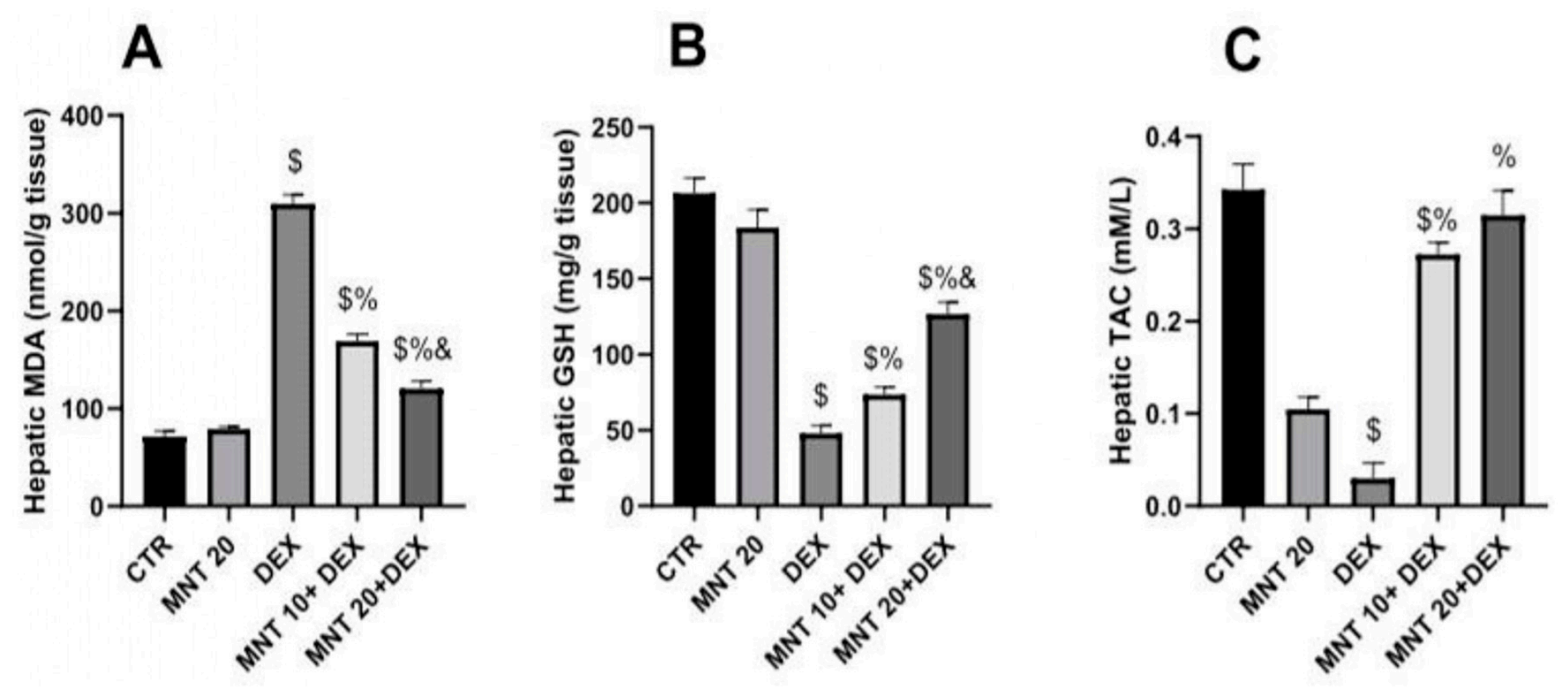
Effect of MNT on DEX-stimulated hepatic oxidative stress. (**A**) MDA, (**B**) GSH, and (**C**) TAC. Data are presented as mean ± SD, *n* = 4; one-way ANOVA was used for analysis, $, %, & *p* < 0.05 compared to the CTR, DEX, and MNT 10 + DEX, respectively. CTR, control; DEX, dexamethasone; MDA, malondialdehyde; MNT, montelukast; GSH, reduced glutathione; TAC, total antioxidant capacity (hepatic TAC).

The effects of MNT on the markers of oxidative stress, including MDA, GSH, and TAC, were determined. DEX extensively (*p* < 0.0001) raised the levels of MDA, while concomitantly lowering GSH content (Figure 12A,B). The results also revealed that TAC was markedly decreased (*p* < 0.0001) by DEX-only treatment (Figure 12C). It was observed that, contrary to the DEX group, MNT effectively (*p* < 0.0001) attenuated the formation of oxidative markers as compared to the DEX group (Figure 12A–C). The MDA levels were significantly (*p* < 0.0001) lowered by both doses of MNT, representing the efficient antioxidant effects of MNT (Figure 12A). Similarly, the antioxidant status was improved by MNT through upregulation of GSH and TAC levels (Figure 12B,C). Interestingly, MNT administration alone (20 mg/kg) was associated with a slight numerical reduction in hepatic TAC compared with the control group (Figure 12C). While this alteration did not reach statistical significance, it warrants further investigation to evaluate potential baseline redox adaptations during high-dose monotherapy. Hence, MNT demonstrated protective impacts against DEX-related oxidative damage.

### 2.13. Influences of Montelukast (10 and 20 mg/kg) on NLRP3, LTD4, and CysLT2 Levels

**Figure 13:**
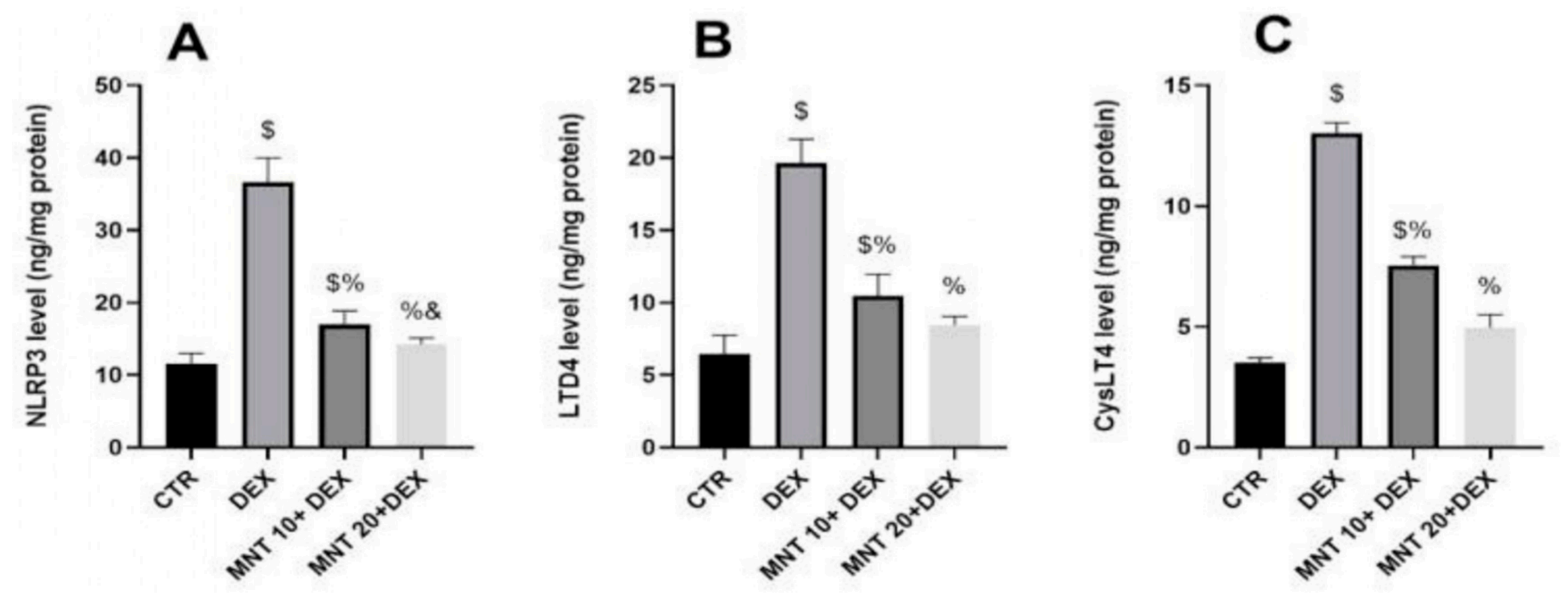
Influence of MNT on (**A**) NLRP3; (**B**) LTD4; (**C**) CysLT2. Data are conveyed as mean ± SD, *n* = 4. $, %, & *p* < 0.05 vs. CTR, DEX, MNT 10 + DEX, correspondingly, via one-way ANOVA. CTR: control; CysLT2: cysteinyl leukotriene 2; DEX: dexamethasone; LTD4: leukotriene D4; MNT: montelukast; NLRP3: NOD-like receptor pyrin domain-containing protein 3.

Dexamethasone administration markedly activated the inflammasome pathway, as evidenced by extensively elevated hepatic levels of NLRP3 compared with the control group (Figure 13A). Pretreatment with MNT at 10 mg/kg significantly lowered NLRP3 expression comparative to the DEX group, whereas MNT at 20 mg/kg produced a more pronounced suppression, with NLRP3 levels significantly (*p* < 0.0001) lower than both DEX and MNT 10 + DEX groups. Similarly, DEX led to a significant (*p* < 0.0001) elevation in LTD4, and CysLT2 levels compared with control animals (Figure 13B,C). Montelukast pretreatment at 10 mg/kg significantly (*p* < 0.0001) reduced both mediators compared with DEX, while the 20 mg/kg dose produced an even greater reduction, achieving a significant (*p* < 0.0001) differences from DEX and being significantly lower than MNT 10 + DEX. Collectively, these findings indicate that MNT effectively attenuates DEX-induced activation of the leukotriene inflammasome axis in a dose-dependent manner.

## 3. Discussion

The current study explored the possibility of MNT protection against DEX-induced metabolic and hepatic dysfunction. DEX administration resulted in hyperglycemia, hyperinsulinemia, increased surrogate indices of insulin resistance, dyslipidemia, hepatic injury, oxidative stress, and inflammatory responses. MNT co-treatment alleviated many of these alterations and was associated with modulation of selected metabolic, insulin-signaling-related, oxidative, and inflammatory markers. These findings provide an integrated characterization of MNT-associated effects in a short-term experimental rat model of glucocorticoid-associated metabolic dysfunction.

An important consideration is the preventive nature of the present design. Montelukast was administered for seven days before DEX exposure and continued throughout DEX treatment; therefore, the study evaluated attenuation of developing DEX-associated metabolic and hepatic abnormalities rather than reversal of established insulin resistance or hepatic injury.

The DEX regimen (8 mg/kg/day, i.p., for seven days) was selected based on experimental evidence showing that this short-term exposure produces a pronounced metabolic–hepatic phenotype in male Wistar rats, including insulin resistance, dyslipidemia, oxidative stress, hepatocellular injury, and steatotic changes. However, the relatively high-dose, short-duration regimen represents an experimental model of acute and pronounced glucocorticoid-associated dysfunction and may not reproduce the magnitude or temporal pattern of conventional clinical glucocorticoid exposure. This should therefore be considered when extrapolating the findings to humans. Similarly, the MNT doses represent experimental rat doses and should not be directly equated with clinical human exposure because of interspecies differences in pharmacokinetics and systemic drug exposure.

In the current model, DEX administration led to pronounced increases in serum ALT and AST levels indicative of hepatocellular injury [20]. These biochemical changes were attenuated by MNT, accompanied by improvement in hepatic histopathological and ultrastructural alterations. DEX also significantly increased TC, LDL-C, Ox-LDL, TG, and FFA, indicating lipid and metabolic dysregulation, whereas these alterations were attenuated by MNT treatment. Importantly, FFA and Ox-LDL in the present study were assessed in hepatic tissue homogenates rather than in the circulation and therefore should be interpreted as hepatic tissue-associated measurements rather than circulating concentrations. The Ox-LDL reduction is especially apropos in the context of increased oxidative stress noted in the current model. Crucially, these results were interpreted in the context of the biochemical and structural data rather than being taken as direct evidence for one single lipid-regulatory mechanism. A similar comparison is provided by Tan et al. who showed that not only was astaxanthin attenuated by MC-LR-induced hepatic lipid metabolic dysfunction but also hepatic triglycerides, total cholesterol, AST, ALT, lipid accumulation, and inflammatory changes were lower together with reduced mRNA transcription levels of Srebp-1c, Fasn, Cd36, Scd1, Dgat1, and Pparg [21]. MNT in the present study likewise ameliorated DEX-induced dyslipidemia and hepatic damage in parallel with improved oxidative/inflammatory parameter status. Nevertheless, the experimental models and pharmacological mechanisms were largely distinct, and this study did not analyze the entire panel of lipogenic genes examined by Tan et al. Consequently, we suggest that the evident lipid improvement should not be viewed as direct modulation of these lipogenic pathways per se. Instead, it may represent the concurrent amelioration of metabolic, oxidative, inflammatory, and insulin-related abnormalities.

Metabolic dysfunction induced by DEX was accompanied by the presence of hyperglycemia and hyperinsulinemia as well as an increase in HOMA2-IR, confirming that glucose homeostasis is impaired with reduced insulin sensitivity indicated from elevated surrogate indices of insulin resistance [22]. MNT reduced these abnormalities and potently improved glucose management. These results may be considered as indicating improved glucose homeostasis and surrogate markers of insulin resistance, rather than measurements of the direct restoration of insulin sensitivity because this type of study did not obtain any data from a hyper-insulinemic-euglycemic clamp for quantitative estimation. In a similar manner to the OGTT, we interpret findings as the AUC and post-glucose stimuli using individual glucose responses rather than a direct measure of insulin sensitivity.

To explore molecular correlates of these metabolic effects, selected insulin-signaling-related proteins and phosphorylation-associated signaling components were examined. This DEX-induced metabolic dysfunction was accompanied by alterations in p-AMPK, p-PI3K, and p-ERK1/2 signals, which were improved by MNT. Changes in phosphorylation tell us about the phosphorylated state of these signaling components and may contribute to changes in pathway signaling. But on their own, they do not provide insight into functional pathway activity. Similarly, the observed changes in total AKT, JNK, IRS-1, and GLUT4 abundance provide information on protein expression but do not establish AKT activation, IRS-1 phosphorylation, or GLUT4 membrane translocation. In particular, hepatic GLUT4 abundance should be interpreted cautiously because hepatic glucose transport is predominantly mediated by GLUT2, while canonical insulin-responsive GLUT4 trafficking is principally described in skeletal muscle and adipose tissue. Since GLUT4 was assessed in whole-liver homogenates, the present data cannot establish hepatocyte-specific expression or membrane localization. GLUT2 abundance and direct measures of hepatic glucose production or utilization were not evaluated. Therefore, the observed changes in hepatic GLUT4 should be regarded as an associated molecular finding rather than evidence of enhanced hepatic glucose uptake through GLUT4. In the present study, we did not measure isoform-specific phosphorylation of AKT1/AKT2, IRS-1 phosphorylation, GLUT4 translocation, or downstream functional glucose uptake [23]. Thus, the detected goings on must be seen as modulation of individual insulin-signaling-related factors and not restoration of whole pathway signaling. Still, however, the inhibition of DEX-induced changes in JNK and IRS-1 by MNT is consistent with modulation of insulin-resistance-associated signaling pathways [24].

AMPK is a master regulator of cellular energy homeostasis and hepatic lipid metabolism [25]. DEX decreased p-AMPK and MNT raised p-AMPK to near control levels in the present study. These findings are consistent with modulation of AMPK-associated energy-regulatory signaling, although functional pathway activation was not directly established. MNT also attenuated DEX-associated changes in the hepatic abundance of the glucose-metabolism-related proteins PEPCK and GSK [26]. Moreover, MNT attenuated the DEX-associated increase in hepatic mTOR immunoreactivity. Since total mTOR expression (rather than mTOR phosphorylation or downstream activity) was examined, these findings would more appropriately be characterized as modulation of AMPK/mTOR-associated signaling rather than direct evidence for pathway activation/inhibition. Similarly, even though AMPK signaling is intimately associated with fatty-acid utilization, this functional effect was not directly evaluated in the current study. The phospho-AMPK findings should also be interpreted with caution. Although the Western blot analysis detected the phospho-AMPK-associated signal using the anti-phospho-AMPK alpha-1 (Thr198) antibody, normalization to β-actin alone does not distinguish changes in AMPK phosphorylation from alterations in total AMPK abundance. In the present study, AMPK protein was additionally assessed by ELISA; however, a matched total-AMPK Western blot was not performed in the same samples and therefore a phospho-AMPK/total-AMPK ratio could not be determined. Accordingly, the Western blot findings should not be regarded as definitive evidence of AMPK activation. Rather, they indicate an alteration in the phospho-AMPK-associated signal under the experimental conditions. Direct assessment of phospho-AMPK and total AMPK in parallel Western blots, with calculation of the corresponding phosphorylation ratio, would provide stronger evidence regarding AMPK phosphorylation status and pathway activation.

The coordinated metabolic phenotype was further associated with changes in the ERK/PI3K/FOXO3/PPARγ signaling network. MNT decreased the DEX-mediated changes in p-ERK1/2 and p-PI3K, which were accompanied with normalization of FOXO3 and PPARγ expression. FoXO3 is involved in the transcriptional regulation of genes implicated in hepatic glucose production and cellular stress response, while PPARγ is a key regulator of lipid metabolism and insensitivity to insulin [27]. While these results lend a potential molecular explanation for the metabolic alterations that have been observed, they do not indicate whether MNT directly targets each element or build a conclusive linear signaling cascade between these proteins. Rather, the observed changes may represent coordinated modulation of interconnected metabolic signaling processes. It is important to distinguish between the PI3K measurements obtained by ELISA and Western blot analysis in the present study. Although both assays refer to PI3K, they do not represent identical molecular readouts. The ELISA measurement reflects the PI3K protein detected by the corresponding immunoassay, whereas the Western blot analysis was performed using a phospho-PI3K p85 alpha-specific antibody (Cat. No. PA5-104853, Thermo Fisher Scientific/Invitrogen, Waltham, MA, USA) and therefore reflects the phosphorylated PI3K p85 alpha species recognized by this antibody. Consequently, differences in the direction or magnitude of the PI3K signals obtained by the two methods should not be interpreted as contradictory findings. Rather, the observed discrepancy may reflect differences between total/detected PI3K protein abundance and the phosphorylated PI3K species assessed by Western blot. Importantly, the present data do not allow a direct quantitative comparison between the two assays or definitive conclusions regarding overall PI3K pathway activation. Further studies assessing total PI3K together with site-specific phospho-PI3K and their corresponding phosphorylation ratios under identical experimental conditions would provide a more comprehensive evaluation of PI3K signaling.

The normalization of metabolic abnormalities was associated with suppression of oxidative and inflammatory mediators. MNT attenuated DEX-associated oxidative stress, as reflected by reduced MDA and improvement in the depleted antioxidant parameters GSH and TAC, suggesting favorable effects on the hepatic redox environment. MNT is primarily characterized as a selective CysLT1 receptor antagonist, and modulation of cysteinyl leukotriene-related signaling may therefore provide biological context for its anti-inflammatory effects [28]. In agreement, MNT inhibited the DEX-stimulated NF-κB and IL-6 responses. Oxidative and inflammatory processes can be amplified during metabolic and hepatic stress, but no temporal or causal hierarchy was inferred in the present study. Thus, these antioxidant effects should be regarded as an additional effect of MNT response and not specifically categorized as downstream to this anti-inflammatory activity.

The relationship between leukotriene signaling and metabolic dysfunction provides additional biological context. Experimental evidence indicates that LTB4 can interfere with insulin-mediated signaling, including reduced insulin-stimulated Akt phosphorylation and increased serine phosphorylation of IRS-1/2, while genetic or pharmacological inhibition of LTB4R1/BLT1 has been associated with improved insulin sensitivity and reduced hepatic steatosis in experimental models [29,30]. These data provide evidence for an LTB4–BLT1 role in the development of metabolic inflammation and insulin resistance. However, BLT1 is different than the CysLT1 receptor targeted by MNT. Inhibiting the LTB4–BLT1 axis as a mechanism of MNT action is not established in the present study, which did not measure LTB4 synthesis, BLT1 expression, or activity directly.

Current results also demonstrated other changes in alterations in the leukotriene ligand LTD4, the CysLT2 receptor, and NLRP3, which were attenuated by MNT treatment. These results are compatible with modulation of cysteinyl leukotriene-related inflammation and NLRP3-mediated responses. Notably, MNT is mainly characterized as a selective CysLT1 receptor antagonist, while in the current study, we evaluated CysLT2. Thus, alteration of CysLT2 receptor abundance should not be interpreted as evidence of direct MNT–CysLT2 antagonism or direct engagement of CysLT2 by MNT. Likewise, downstream of inflammasome cleavage products, like caspase-1 and IL-1β, were not directly measured, so these data should be interpreted with caution regarding direct evidence that inhibition or activation of the inflammasome occurred in this model. Previous studies reporting attenuation of leukotriene-associated inflammatory and NLRP3-related responses following MNT treatment provide complementary biological context [18,31,32,33].

Previous study indicated that Lactobacillus reuteri prevented methotrexate-induced hepatic injury by modulating oxidative stress and inflammatory signaling (HO-1/GPX4 and NF–κB/NLRP3) [34]. This example gives an idea about the convergence of various hepatoprotective interventions on oxidative/inflammatory processes. However, the current study is quite different regarding pharmacological intervention and disease model; MNT is a CysLT1 receptor antagonist, and this model obeys DEX-induced metabolic dysfunction. Thus, the comparison is more mechanistic than evidence of similar pharmacological action.

Another point to consider is the paradoxical increase in hepatic IL-6 and NLRP3 after exposure to DEX. Known for its powerful anti-inflammatory properties, DEX has been shown to inhibit proinflammatory mediators and signals such as IL-6 and NLRP3 in specific experimental settings [35,36]. Glucocorticoid effects can vary according to tissue, dose, duration, and metabolic context [37,38]. In this model, DEX was linked to insulin resistance, hyperglycemia, dyslipidemia, oxidative stress, and hepatic injury consistent with a metabolic and redox milieu associated with the observed inflammatory phenotype. This interpretation is still conjectural since the present study did not directly address mechanisms underlying the observed inflammatory phenotype.

The distinction between an inflammatory biomarker and a functionally relevant mediator is also important. Mu et al., in an unrelated aging model, combined assessment of TNF-α with neutralization and supplementation experiments to provide functional evidence for the contribution of this inflammatory mediator to the observed phenotype. Although their model involved gut microbiota-related neuroinflammation rather than glucocorticoid-induced hepatic metabolic dysfunction, the study illustrates how targeted perturbation experiments can strengthen causal interpretation beyond changes in biomarker abundance alone. In the present study, NLRP3 abundance and inflammatory mediators were assessed without direct perturbation or direct functional interrogation of NLRP3 inflammasome assembly or activation; therefore, these findings should be interpreted as changes in inflammasome-associated inflammatory markers rather than definitive evidence of inflammasome activation or causality [39].

Likewise, SGK1 and NLRP3 signaling should also be cautiously interpreted in relation to each other. Despite previous reports suggesting that SGK1 may modulate NLRP3-associated inflammatory responses in specific experimental settings, the current study demonstrated a concomitant increased hepatic SGK1 and NLRP3 following DEX treatment [40,41]. This may reflect context-dependent regulation, or a compensatory response to metabolic or oxidative stress [41,42], but this possibility cannot be confirmed by the present design. No pharmacological inhibition, genetic manipulation, direct protein–protein interaction analysis, or direct SGK1 kinase activity assay was performed. Thus, reduced SGK1 and NLRP3 following MNT is associated with an overarching response of metabolic and inflammatory changes rather than establishing a direct causal relationship between SGK1 and NLRP3-associated responses.

The distinction between molecular association and causal mechanism is important when interpreting the present findings. Chen et al., using an unrelated viral infection model, combined multi-omics analyses with target-directed functional experiments to identify the CRYAB–RBM26 axis underlying curcuminoid-associated antiviral responses [43]. Although this biological context is distinct from the present study, it provides a useful methodological example of how functional perturbation of candidate targets can strengthen mechanistic inference. In the present model, such target-directed approaches, including pharmacological or genetic manipulation of SGK1, would be required to determine whether SGK1 causally contributes to the metabolic and hepatic effects associated with MNT rather than changing in parallel with these outcomes.

In summary, the simultaneous improvement in glucose homeostasis, lipid abnormalities, oxidative status, inflammatory markers, and serum hepatic enzymes as well as histopathological changes suggest that the metabolic improvements associated with MNT treatment may have contributed to the attenuation of liver injury. Collectively, the coordinated improvement in metabolic, oxidative, inflammatory, and hepatic parameters, together with changes in selected signaling-related proteins, provides an integrated context for the effects of MNT in this model. These molecular alterations should, however, be regarded as treatment-associated changes rather than evidence of a defined linear signaling cascade or direct pathway activation. 

**Figure 14:**
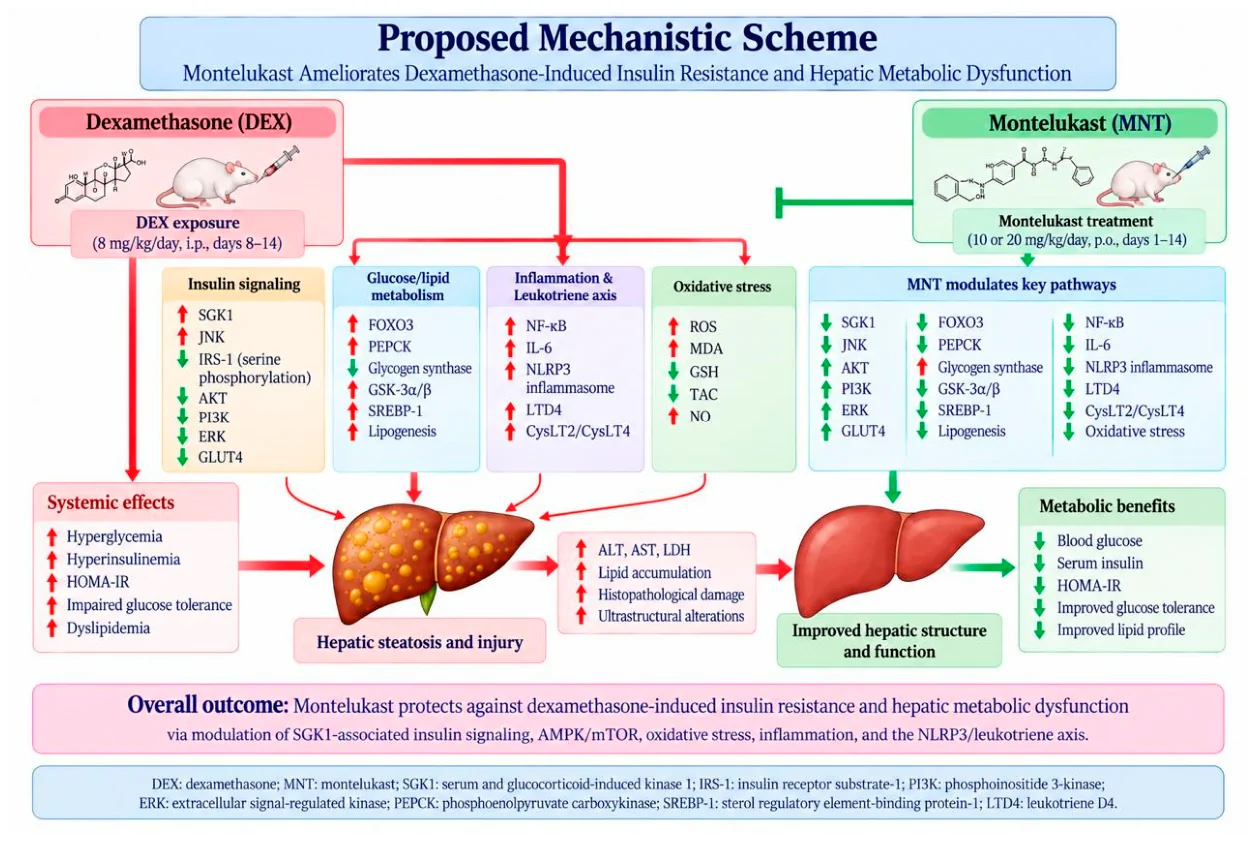
Proposed mechanistic framework for the known mechanisms that could explain the protective effects of montelukast (MNT) against dexamethasone (DEX)-induced metabolic and hepatic dysfunction. The diagram illustrated the role of DEX-induced metabolic stress in that citated changes in SGK1-associated insulin signaling, ERK/PI3K-related signaling, FOXO3/PPARγ and AMPK/mTOR-associated energy regulation products together with dyslipidemia, oxidative stress, and inflammatory activation. These metabolic, oxidative, inflammatory, and hepatic abnormalities were blunted with MNT treatment along with modulation of the signaling components we investigated. Schematic drawing of molecular interaction pathways within our proposed integrative model based upon the data generated in the present study and literature to date, although future comprehensive studies will undoubtedly reveal many more. The schematic does not suggest a linear or deterministic relationship between SGK1 and the ERK/PI3K/FOXO3/PPARγ pathway as neither pathway-specific inhibition nor genetic manipulation, direct protein–protein interaction studies, or functional pathway assays were undertaken.

The proposed mechanistic framework for the known mechanisms that could explain the protective effects of montelukast (MNT) against dexamethasone (DEX)-induced metabolic and hepatic dysfunction is shown in Figure 14.

## 4. Limitations

Several limitations should be acknowledged. The DEX-induced model also has some important limitations. First, it represents an isolated pharmacologically induced form of glucocorticoid-mediated metabolic dysregulation and does not fully recapitulate the complex pathophysiology of human metabolic disease. The short course and high levels of DEX used in non-human subjects also restrict direct clinical translation. Secondly, only male rats were used which reduces generic applicability due to a potential influence of sex-dependency differences affecting glucocorticoid responses, insulin sensitivity, lipids metabolism, and inflammatory signaling. Third, a limitation of the present study is the absence of an MNT10-alone group and the restriction of Western blot analysis to the CTR, DEX, and MNT20 + DEX groups. Therefore, the intrinsic effects of MNT10 and the dose-dependent effects of MNT on the examined signaling markers cannot be fully determined. Future studies including both MNT10- and MNT20-alone groups and all treatment groups in Western blot analyses would provide a more comprehensive dose–response assessment. Pharmacokinetics of MNT should also be investigated when both doses are used as monotherapy, in both sexes and against proper reference hepatoprotective treatment in future studies. Fourth, the study was limited by a focus on targeted biochemical and molecular measurements rather than comprehensive metabolomic profiling. However, HbA1c was not measured, and longer-term glycemic exposure could not therefore be assessed. While selected signaling components were assessed, direct pathway activity was not validated. Furthermore, the PI3K measurements obtained by ELISA and Western blot represent different molecular readouts, as the ELISA assessed PI3K immunoreactivity whereas the Western blot evaluated phospho-PI3K p85 alpha. Therefore, these measurements cannot be directly compared quantitatively, and the observed findings should not be interpreted as definitive evidence of overall PI3K pathway activation. Assessment of total PI3K, site-specific phospho-PI3K, and their corresponding phosphorylation ratios in the same experimental groups would provide a more comprehensive characterization of PI3K signaling. In addition, the observed changes in total hepatic AKT and IRS-1 abundance do not, by themselves, define signaling activity or functional insulin signaling, while changes in total GLUT4 abundance cannot establish its translocation to the plasma membrane. Hepatic GLUT4 findings require cautious interpretation because GLUT4 is not the predominant glucose transporter responsible for hepatocyte glucose uptake. GLUT2 was not assessed, and the use of whole-liver homogenates did not permit determination of the cellular source or membrane localization of the GLUT4 signal. Direct measurements of hepatic glucose production, glucose utilization, or tissue-specific glucose uptake were also not performed. Therefore, the present findings cannot establish a GLUT4-mediated improvement in hepatic glucose handling. Moreover, although AMPK protein abundance was assessed by ELISA and a phospho-AMPK-associated signal was evaluated by Western blot, a matched total-AMPK Western blot was not available for normalization of the phospho-AMPK signal. Consequently, the present data do not establish changes in AMPK phosphorylation or activation independently of total protein abundance. Future studies incorporating paired phospho-AMPK and total-AMPK Western blot analyses and calculation of the phospho/total ratio would provide more definitive evidence of AMPK pathway activation. Thus, the present findings should be interpreted as alterations in selected insulin-signaling-related components rather than direct evidence of enhanced or impaired pathway activity. Functional evidence would be strengthened by direct assessment of phospho-AKT isoforms and IRS-1 phosphorylation, GLUT4 membrane translocation, relevant kinase activity, and downstream inflammasome cleavage products. These additional assessments would help determine whether the observed molecular changes translate into functional alterations in insulin signaling and inflammasome activation. Furthermore, food consumption was not quantitatively monitored in the present study; therefore, its potential contribution to the observed metabolic and biochemical changes could not be directly assessed. Last but not least, DEX and MNT treatment-affected SGK1 immunoreactivity dependence on SGK pathway was not proven since neither pharmacological inhibition nor genetic manipulation of SGK1 were performed and its kinase activity was never directly measured. Consequently, changes in SGK1 should be understood as being positively correlated with the metabolic and hepatic phenotype observed, rather than evidence that SGK1 is essential for at least some of the consequences of MNT. The mechanism plays a role that can only be interrogated in future pathway-specific intervention studies.

## 5. Translational Medicine

The translational relevance of these findings should be considered within the specific context of glucocorticoid-associated metabolic and hepatic dysfunction. In this short-term preventive rat model, MNT attenuated several DEX-associated abnormalities, including impaired glucose homeostasis, dyslipidemia, oxidative stress, inflammatory responses, and hepatic injury. These findings provide experimental support for further investigation of leukotriene-related signaling in glucocorticoid-associated metabolic dysfunction. However, the study was designed as a preventive/protective model rather than a therapeutic model of established insulin resistance; therefore, the findings do not establish reversal of established insulin resistance or clinical hepatoprotection in humans.

The high pharmacological DEX exposure, short treatment duration, male-only design, absence of comprehensive pharmacokinetic assessment, absence of a reference hepatoprotective treatment, and lack of pathway-specific intervention further limit direct translation to clinical practice. Future studies should evaluate longer-term glucocorticoid exposure in both sexes, together with dose–response, pharmacokinetic, and safety assessments. Direct investigation of leukotriene-related pathways and pathway-specific interventions will also be required to clarify the mechanistic basis of the observed effects. Ultimately, appropriately designed clinical studies are necessary to determine whether the experimental effects of MNT can translate into clinically meaningful benefits in glucocorticoid-associated metabolic and hepatic dysfunction.

## 6. Materials and Methods

### 6.1. Drugs and Chemicals

DEX obtained from Amriya Pharmaceutical Industries (8 mg/2 mL, USP^®^, Alexandria, Egypt) was diluted in sterile 0.9% normal saline solution immediately prior to injection. MNT (Asmakast^®^ tablets, 10 mg, film-coated; Sandoz, Princeton, NJ, USA), for oral administration by oral gavage, was freshly suspended in carboxymethylcellulose (CMC; a 0.5% *w*/*v*).

### 6.2. Animals

In the current study, adult male Wistar rats weighing 200 ± 20 g were obtained from Vacsera (Giza, Egypt). Upon arrival, the animals were acclimatized for one week under standard laboratory conditions at a temperature of 25 ± 2 °C, with a 12 h light/dark cycle and relative humidity of approximately 50–60%. Rats were housed in groups of 3–4 animals per cage with free access to standard laboratory chow and water ad libitum. The standard chow consisted of 54% carbohydrates, 26% protein, 17% minerals and vitamins, and 3% fat. Only healthy animals within the predefined body-weight range and without apparent signs of illness or abnormality were included in the study. Each animal was assigned a unique identification number before experimental allocation to allow individual tracking throughout the study. Animals were acclimatized before allocation and treatment initiation. The animal study protocol was approved by the Mansoura University Animal Care and Use Committee (MU-ACUC), Faculty of Pharmacy Scientific Research Ethics Committee (protocol code: PHARM. PhD.24.06.43). All animal experiments were conducted in accordance with the Guide for the Care and Use of Laboratory Animals, 8th edition, National Research Council, USA, 2011. The study was performed in accordance with the ARRIVE guidelines.

### 6.3. Animal Allocation, Endpoint-Specific Sample Sizes, and Experimental Unit

In this study, male Wistar rats (*n* = 30) were assigned to five experimental groups (*n* = 6 per group) through a stratified randomization based on the initial body weight. In brief, all animals were weighed and screened for eligibility prior to allocation into groups and allocated to one of five groups such that mean initial body weights did not differ significantly between the groups, thus minimizing potential selection bias. Baseline blood glucose measurements and other metabolic challenge tests (including an oral glucose tolerance test) were not performed prior to randomization into groups. Thus, allocation was determined by established eligibility criteria and initial body weight rather than glycemic or glucose-tolerance responses at baseline.

Of the 30 animals allocated, all completed the experimental protocol without loss to follow-up. Six animals were initially allocated to each group, and all 30 animals completed the experimental protocol. For individual analytical endpoints, however, subsets of these allocated animals contributed biological samples according to the requirements of the respective assays. The biological sample size for each endpoint is therefore stated explicitly within the figure legend associated with each figure. If *n* = 4 or *n* = 3 is reported, this indicates the number of independent animals contributing biological samples to that specific analysis and does not represent post hoc exclusion based on experimental findings.

For Western blot, (*n* = 3) represents three biologically independent animals per indicated group. Technical measurements, replicate wells, or repeated measurements from an individual animal were not counted as independent biological replicates. In addition, if more than one tissue section or microscopic field was selected for histopathological or immunohistochemical assay, then these observations were recorded as subsampling measurements (not as independent experimental units) from the same animal. Statistical analyses were performed on animal-level values.

Subsets of animals for individual analysis endpoints were selected according to the experimental sampling and assay requirements, not based on significance or magnitude of findings. There were no outcome-dependent exclusion and animals were not selectively removed. The exclusion criterion was predefined as the inability to fulfill the eligibility criteria of the experiment before random assignment; no animal in either group met exclusion criteria post random assignment. Therefore, all animals completing the experimental protocol remained eligible for planned outcomes.

### 6.4. Randomization, Allocation, and Blinding

Randomization was performed before treatment initiation using stratified allocation based on initial body weight. The previous wording “haphazardly distributed” has therefore been removed and replaced with this precise description of the allocation procedure. Allocation concealment was not formally implemented because the treatment assignments were established according to the predefined experimental design. Treatment administration was not performed under a formal blinded protocol. However, histopathological and immunohistochemical assessments were performed by experienced observers blinded to experimental group identity. Image-based quantification was conducted according to predefined analytical procedures while the observers remained blinded to group allocation. Biochemical and molecular assays were conducted according to predefined experimental procedures and standardized analytical methods; formal blinding was not implemented for these laboratory assays.

### 6.5. Sample-Size Determination

Before the commencement of our experiment, sample size was determined by using the resource equation approach for exploratory animal study [44,45]. Thus, six animals were used for each experimental group. The selected group size was more in line with our previous study using a similar dexamethasone-induced metabolic model [46] while taking into account the 3R principles by reducing the use of animals. No formal a priori statistical power calculation on an expected effect size was undertaken. Thus, the current study was designed as exploratory and mechanistic animal research, not formally powered efficacy studies.

Throughout the study, animals were regarded as the biological experimental unit rather than an individual microscopic field, technical replicate, or repeated measurement. Detailed in the figure legends are the total number of independent animals that contributed to each endpoint.

### 6.6. Exclusion Criteria

Animals that did not meet the prespecified eligibility criteria would have been excluded before randomization; however, no animal met these exclusion criteria. No post-allocation exclusions were applied.

### 6.7. Study Design

**Figure 15:**
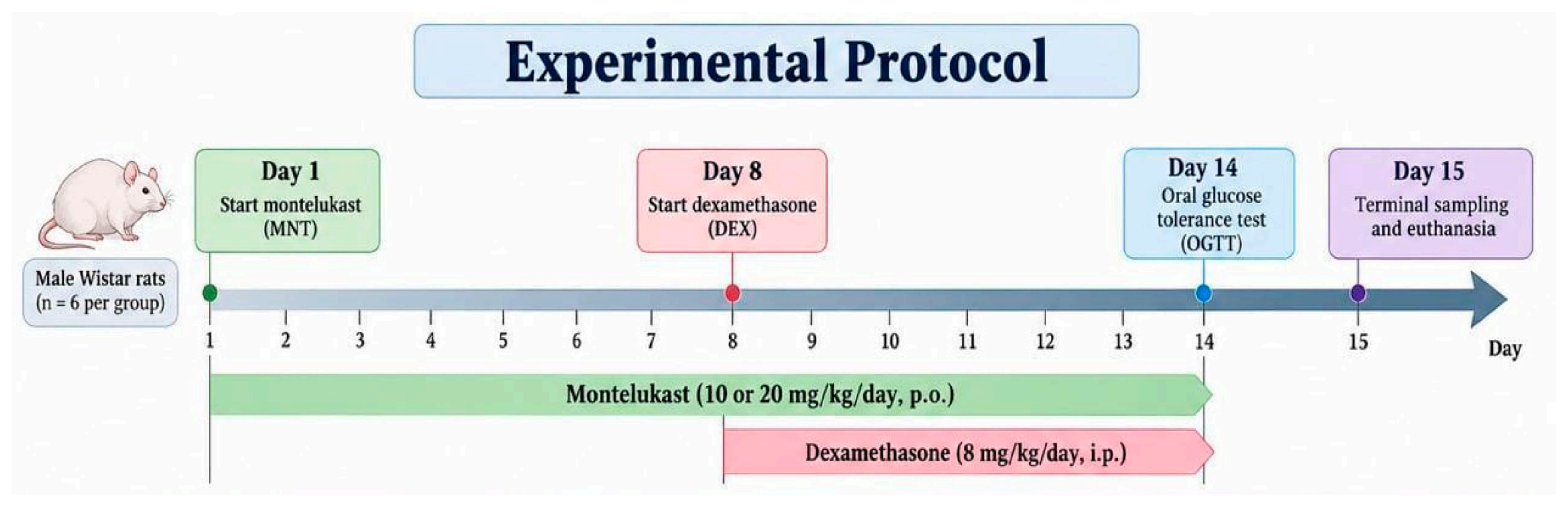
Design of experimental protocol.

The experimental groups were as follows: Control group: Rats left untreated for 14 consecutive days (received only vehicles). MNT20 group: Rats received MNT alone (20 mg/kg/day, orally) for 14 days [47]. DEX group: Rats were injected with DEX (i.p., 8 mg/kg/day, 7 days) starting on day 8 of the experiment [46]. MNT10 + DEX group: Rats were pretreated orally with MNT (10 mg/kg/day) for 14 days [19,48]. On day 8, DEX application started. MNT20 + DEX group: Rats were pretreated with MNT (20 mg/kg/day/orally/14 days) [47] and started DEX on day 8, which was injected one hour after MNT administration (Figure 15).

The MNT doses of 10 and 20 mg/kg/day were selected based on previously reported effective experimental doses in rodent models. In particular, the 20 mg/kg/day dose was selected based on the study by Hegab et al. who reported protective effects of MNT at this dose against prednisolone-induced hepatic injury in rats [47]. The 10 mg/kg/day dose was included as a lower experimental dose to provide a dose-ranging comparison. These doses were not intended to represent ED50 values, human-equivalent therapeutic doses, or formally optimized doses, but rather experimentally relevant doses for evaluating the preventive/protective effects of MNT in the present model.

The DEX dose of 8 mg/kg/day was selected based on previously reported experimental protocols to establish a robust and reproducible glucocorticoid-induced metabolic phenotype within a short exposure period; 7 days [6,49]. This dose represents a high pharmacological experimental exposure and was not intended to quantitatively mimic a therapeutic human DEX dose. Therefore, direct dose-to-dose extrapolation to clinical exposure is not appropriate.

Male Wistar rats were selected based on the established use of this species for experimental induction of glucocorticoid-associated metabolic dysfunction by dexamethasone, including hyperglycemia, impaired glucose tolerance, hyperinsulinemia/insulin resistance, and hepatic metabolic abnormalities.

The experimental treatment schedule was designed as a preventive/protective paradigm. MNT administration was initiated before DEX exposure and continued throughout the experimental period, whereas DEX administration was initiated on day 8. This sequence was selected to evaluate whether MNT could attenuate the development of DEX-associated metabolic and hepatic disturbances during glucocorticoid exposure. Therefore, the present design was intended to assess preventive/protective efficacy rather than reversal of an already established state of insulin resistance.

Body weights were recorded repeatedly, and animals were monitored for signs of distress throughout the study period. At the completion of the experiment, the individual weights of rats were measured, and the blood samples were removed from the retro-orbital venous plexus using light anesthesia with secobarbital (50 mg/kg, i.p., 1st step euthanasia step). The blood samples were kept for a while to clot, then centrifuged (3000 rpm, 15 min at 4 °C) to extract serum that was kept at −20 °C for subsequent biological evaluations. Rats were humanely euthanized by cervical dislocation for tissue collection after completing blood gathering (2nd step euthanasia process). Liver tissues were promptly excised, rinsed to remove any blood with ice-cold saline, blotted dry, and weighed on a digital balance. Each liver tissue was sectioned into two parts: one part was fixed in neutral-buffered formalin (10%) for different histopathological analysis, and the other portion was kept at −80 °C for biochemical analyses and tissue homogenate preparation.

### 6.8. Oral Glucose Tolerance Test (OGTT)

On day 12 of the experimental protocol, an oral OGTT was performed to assess glucose homeostasis in all experimental groups. Rats were overnight fasted with free access to water before the test. Each conscious rat was then orally administered a 40% glucose solution at a dose of 2 g/kg body weight using an oral gavage tube [49]. Tail-vein blood samples were collected immediately before glucose administration (0 min) and at 30, 60, 120, and 180 min after glucose loading. Blood glucose concentrations were measured using an Accu-Chek^®^ glucometer (Hoffmann-La Roche Ltd., Basel, Switzerland). The glucose response was further evaluated by calculating the area under the glucose–time curve (AUC) using the trapezoidal rule.

### 6.9. Fasting Serum Glucose and Insulin

Throughout the 14-day experiment time, rats had an ad libitum approach to both standard chow and clean water. On the evening of day 14, animals were fasted for 10 h with continued access to water. Serum insulin levels were quantified using rat-specific insulin ELISA kit (# RTC018R, Biovendor-Laboratorni medicina, Brno, Czeh Republic) following the industrialist‘s procedures. Homeostasis model assessment of insulin resistance (HOMA-IR) values was determined using the HOMA2 model through the HOMA2 Calculator software (Version 2.2.2, Diabetes Trials Unit, University of Oxford, Oxford, UK), based on paired fasting glucose and insulin concentrations. The fasting glucose and insulin values were entered into the calculator using the units required by the HOMA2 model, without applying the conventional HOMA1 conversion formula. The resulting HOMA2-IR values were used as a surrogate index of insulin resistance rather than as a direct measure of pancreatic β-cell function or insulin sensitivity [49].

### 6.10. Histopathology and Immunohistochemistry

To evaluate hepatic pathology, 5 μm paraffin slices of rat liver tissue were processed with standard hematoxylin and eosin (H&E) staining. All histopathological assessments were performed by an observer blinded to the study groups using a light microscope. Histopathological lesions were evaluated semi-quantitatively based on three parameters: hepatocellular degeneration, necrosis, and inflammatory cell infiltration. Each parameter was scored on a scale of 0–3, where 0 = absent, 1 = mild, 2 = moderate, and 3 = severe. The individual scores were summed to generate a cumulative histopathological score ranging from 0 to 9. For each animal, six randomly selected microscopic fields were examined and averaged to obtain the final animal-level score. Six animals were analyzed per group (*n* = 6) as previously described [50]. The protein expression levels of SGK1, mTOR, and SREBP-1 in rat liver were analyzed using immunohistochemical (IHC) techniques. Following standard deparaffinization and rehydration, endogenous peroxidase activity was blocked with 3% H_2_O_2_. Antigen retrieval was performed using citrate buffer (pH 6.0) for 1 h. Primary antibodies were applied at a dilution of 1:100. Sections were then incubated overnight at 4 °C with primary antibodies specific to each target: SGK1 (ELK Biotechnology, catalog no. EM1264), mTOR (ELK Biotechnology, catalog no. ES2865), and SREBP-1 (ELK Biotechnology, catalog no. ES8814). Signal detection was performed using a biotin–streptavidin–horseradish peroxidase (HRP) system with 3,3′-diaminobenzidine (DAB) as the chromogenic substrate. Negative control sections were processed in parallel using the same staining procedure but with omission of the primary antibody to assess non-specific/background staining. The stained sections were examined using an Olympus light microscope.

IHC evaluation was performed by an experienced pathologist who was blinded to the experimental groups. Quantitative analysis was performed using (Image J Fiji, version 1.54p; NIH, Bethesda, MD, USA) by measuring the DAB-positive immune-stained area. Four non-overlapping sections per animal were assessed, with three high-power fields analyzed per section. The results are expressed as the percentage of DAB-positive immune-stained tissue area relative to the total analyzed tissue area, rather than normalized to cell number. 

### 6.11. Transmission Electron Microscopy (TEM)

After 24 h preservation with 2.5% glutaraldehyde and 2.5% paraformaldehyde using phosphate buffer, 1% osmium tetroxide was applied for post-fixing, followed by gradual dehydration and embedding in the resin. Ultrathin liver slices were placed upon copper grids and further stained with uranyl acetate and lead citrate. We tested the prepared slices with a transmission electron microscope (JEOL2100, Tokyo, Japan). Ultrastructural assessment was performed using standardized examination criteria focusing on hepatocellular architecture, mitochondrial integrity, endoplasmic reticulum organization, nuclear morphology, and lipid accumulation. Images were acquired using identical microscope and camera settings for all experimental groups. Six randomly selected non-overlapping fields were examined for each animal. Quantitative TEM analysis was performed using ImageJ/Fiji on six randomly selected fields per animal. The total number of mitochondria, nuclear diameter, and percentage of damaged mitochondria were measured. Damaged mitochondria were identified based on ultrastructural abnormalities such as swelling, cristae disruption, and membrane damage. The percentage of damaged mitochondria was calculated as Damaged mitochondria (%) = (Number of damaged mitochondria/Total mitochondria) × 100. Steatosis was additionally graded according to the percentage of affected hepatocytes using a predefined five-tier scale. [51].

### 6.12. Determination of Liver Biomarkers

Serum levels of alanine aminotransferase (ALT) and aspartate aminotransferase (AST) were detected to assess hepatic function. The enzymatic activities were detected using commercial kinetic assay kits AGAPPE Diagnostics (India) subsequent to the constructer’s directions.

### 6.13. Assessment of Lipid Profile

The serum lipid profile was assessed by enzymatic colorimetric determination of total cholesterol (TC) and triglycerides (TG) using commercial assay kits supplied by Genesis (Obour City, Egypt). HDL-C levels were analyzed with a precipitation-based kit (Biodiagnostic, Cairo, Egypt), whereas LDL-C concentrations were computed via the Friedewald formula (LDL-C = TC − [HDL-C + VLDL-C]) [52], with VLDL-C estimated as TG/5. The calculated LDL-C values were considered estimated concentrations rather than directly measured LDL-C. The use of this calculation in the present rat model was interpreted with appropriate caution in view of species-related differences in lipoprotein metabolism [53,54].

### 6.14. Oxidative Stress Indicators Assessment

Hepatic levels of reduced glutathione (GSH), malondialdehyde (MDA), and total antioxidant capacity (TAC) were quantified via easily obtainable kits (Biodiagnostic, Egypt) according to the manufacturer’s recommended protocols.

### 6.15. Enzyme-Linked Immunosorbent Assay (ELISA)

Supernatants from liver tissues were separated for ELISA analysis by centrifugation. Following the manufacturer’s protocols, the following protein markers were quantified via commercial ELISA kits (Table 1).

### 6.16. Western Blotting Analysis

According to manufacturer’s protocol, Trifast reagent (Peqlab, VWR, Darmstadt, Germany) was used for extraction of total protein, then the protein concentration in each tissue sample was assessed using the Bradford protein assay kit; 30 µg protein per sample was separated by gel electrophoresis on SDS-polyacrylamide gel and then transferred to PVDF membrane (Amersham Hybond, GE10600023, Merk, Darmstadt, Germany). Membrane was blocked with 2–5% nonfat dry milk for 1 h at room temperature and incubated at 4 °C overnight with specific primary antibodies against Phospho-AMPK alpha-1 (Thr198, # BS-5551R-PE, Thermo Fisher Scientific/Invitrogen, Waltham, MA, USA), phospho-PI3K p85 alpha (# PA5-104853, Thermo Fisher Scientific/Invitrogen, Waltham, MA, USA), and phospho-ERK1/ERK2 (Thr202, Tyr204, # 36-8800, Thermo Fisher Scientific/Invitrogen, Waltham, MA, USA). Membrane was incubated for 1 h at room temperature with horseradish peroxidase (HRP)-conjugated secondary antibody (Novus Biologicals, Centennial, CO, USA). β-actin was applied as housekeeping protein. β-actin was used to normalize all target protein bands across all membranes. Protein bands were visualized and quantified using GelDoc-it, UVP, Cambridge, UK) imaging system and TotalLab software (Ver.1.0.1).

### 6.17. Statistical Analysis

The distributional assumptions were assessed before statistical analysis. Normality of the data was evaluated using the Shapiro–Wilk test, while homogeneity of variance was assessed using Levene’s test. Parametric data that met the relevant assumptions are presented as mean ± standard deviation (SD) and were analyzed using one-way or two-way analysis of variance (ANOVA), as appropriate, followed by the Tukey–Kramer post hoc test. When the assumptions required for parametric analysis were not met, data were analyzed using non-parametric methods and are presented as median and interquartile range (IQR). The Kruskal–Wallis test followed by Dunn’s multiple-comparison post hoc test was used for comparisons among multiple groups.

The study included relatively small experimental group sizes (*n* = 4–6 per group). Sample sizes were determined before initiation of the experiment using the resource equation approach, with consideration of the principle of reduction of animal use. No formal a priori power analysis based on an expected effect size was performed. Therefore, the statistical findings were interpreted in the context of the exploratory and mechanistic nature of the study and the relatively small group sizes.

All statistical analyses were performed using GraphPad Prism version 8.0 (GraphPad Software, Inc., San Diego, CA, USA). A *p*-value < 0.05 was considered statistically significant.

The number of animals included in each assay is indicated in the corresponding figure legends.

Where applicable, exact *p*-values are reported in the Section 2 and/or corresponding figure annotations, in addition to the predefined significance threshold.

## Figures and Tables

**Table 1:** Commercially available ELISA kits used.

Marker	Catalog No.	Company (Country)
PI3K	MBS9356979	MyBioSource, San Diego, CA, USA
PPAR-γ	MBS2701257	
NLRP3	MB2033695	
NF-κB p65	MBS7226224	
IRS-1	MBS2706135	
GSK-3α/β	MBS2513979	
ERK	MBS034880	
FOXO3a	MBS9359523	
Free fatty acids (FFA)	MBS733451	
AKT	MBS3807575	
AMPK	ER1877	FineTest, Wuhan, China
JNK	EK306	ELK Biotechnology, Wuhan, China
LTD4	ELK9178	
CysLT2	ELK5634	
GLUT4	CSB-E13908r	CUSABIO, Wuhan, China
Glycogen synthase (GS)	RE2350R	Reed Biotech, Wuhan, China
IL-6	R6000B	R&D Systems, Minneapolis, MN, USA
Ox-LDL	E-CL-R0502	Elabscience, Houston, TX, USA
PEPCK	RAT09161	Assay Genie, Dublin, Ireland

Hepatic Ox-LDL levels were determined using a rat-specific OxLDL CLIA kit (E-CL-R0502, Elabscience, Houston, TX, USA), according to the manufacturer’s instructions. Hepatic tissue homogenates were used for analysis. The obtained values were interpreted as tissue-associated Ox-LDL measurements within the experimental hepatic model and were not considered direct measures of circulating Ox-LDL.

## Data Availability

All data generated and analyzed during the current study are presented in this manuscript.

## Abbreviations

The following abbreviations are used in this manuscript:

Abbreviation	Full Term
AKT	Protein kinase B
ALT	Alanine aminotransferase
AST	Aspartate aminotransferase
CMC	Carboxymethylcellulose
CysLT2	Cysteinyl leukotriene receptor 2
DEX	Dexamethasone
ERK	Extracellular signal-regulated kinase
FFA	Free fatty acid
FOXO3a	Forkhead box O3
GLUT4	Glucose transporter type 4
GS	Glycogen synthase
GSH	Glutathione
GSK-3α/β	Glycogen synthase kinase-3 alpha/beta
HDL-C	High-density lipoprotein cholesterol
HOMA-IR	Homeostatic Model Assessment of Insulin Resistance
IHC	Immunohistochemistry/Immunohistochemical
IL-6	Interleukin-6
IR	Insulin resistance
IRS-1	Insulin receptor substrate-1
JNK	c-Jun N-terminal kinase
LDL-C	Low-density lipoprotein cholesterol
LTD4	Leukotriene D_4_
MAFLD	Metabolic dysfunction-associated fatty liver disease
MDA	Malondialdehyde
MNT	Montelukast
mTOR	Mammalian target of rapamycin
NAFLD	Non-alcoholic fatty liver disease
NF-κB-p65	Nuclear factor kappa-light-chain-enhancer of activated B cells (p65 subunit)
NLRP3	Nucleotide-binding oligomerization domain, leucine-rich repeat and pyrin domain-containing 3
OGTT	Oral glucose tolerance test
Ox-LDL	Oxidized low-density lipoprotein
p-AMPK	Phosphorylated AMP-activated protein kinase
PEPCK	Phosphoenolpyruvate carboxykinase
PI3K	Phosphoinositide 3-kinase
PPAR-γ	Peroxisome proliferator-activated receptor gamma
ROS	Reactive oxygen species
SGK1	Serum/glucocorticoid-regulated kinase 1
SREBP-1	Sterol regulatory element-binding protein-1
TAC	Total antioxidant capacity
TC	Total cholesterol
TEM	Transmission electron microscopy
TGs	Triglycerides
